# Molecular Correlates of Hemorrhage and Edema Volumes Following Human Intracerebral Hemorrhage Implicate Inflammation, Autophagy, mRNA Splicing, and T Cell Receptor Signaling

**DOI:** 10.1007/s12975-020-00869-y

**Published:** 2020-11-18

**Authors:** Marc Durocher, Bodie Knepp, Alan Yee, Glen Jickling, Fernando Rodriguez, Kwan Ng, Xinhua Zhan, Farah Hamade, Eva Ferino, Hajar Amini, Paulina Carmona-Mora, Heather Hull, Bradley P. Ander, Frank R. Sharp, Boryana Stamova

**Affiliations:** 1 Department of Neurology, School of Medicine, University of California at Davis, Sacramento, CA USA; 2 Department of Medicine, University of Alberta, Edmonton, Canada

**Keywords:** Intracerebral hemorrhage volume, Perihematomal edema volume, Inflammation, Volume, Hematoma clearance, Gene expression

## Abstract

Intracerebral hemorrhage (ICH) and perihematomal edema (PHE) volumes are major determinants of ICH outcomes as is the immune system which plays a significant role in damage and repair. Thus, we performed whole-transcriptome analyses of 18 ICH patients to delineate peripheral blood genes and networks associated with ICH volume, absolute perihematomal edema (aPHE) volume, and relative PHE (aPHE/ICH; rPHE). We found 440, 266, and 391 genes correlated with ICH and aPHE volumes and rPHE, respectively (*p* < 0.005, partial-correlation > |0.6|). These mainly represented inflammatory pathways including NF-κB, TREM1, and Neuroinflammation Signaling—most activated with larger volumes. Weighted Gene Co-Expression Network Analysis identified seven modules significantly correlated with these measures (*p* < 0.05). Most modules were enriched in neutrophil, monocyte, erythroblast, and/or T cell-specific genes. Autophagy, apoptosis, HIF-1α, inflammatory and neuroinflammatory response (including Toll-like receptors), cell adhesion (including MMP9), platelet activation, T cell receptor signaling, and mRNA splicing were represented in these modules (FDR *p* < 0.05). Module hub genes, potential master regulators, were enriched in neutrophil-specific genes in three modules. Hub genes included NCF2, NCF4, STX3, and CSF3R, and involved immune response, autophagy, and neutrophil chemotaxis. One module that correlated negatively with ICH volume correlated positively with rPHE. Its genes and hubs were enriched in T cell-specific genes including hubs LCK and ITK, Src family tyrosine kinases whose modulation improved outcomes and reduced BBB dysfunction following experimental ICH. This study uncovers molecular underpinnings associated with ICH and PHE volumes and pathophysiology in human ICH, where knowledge is scarce. The identified pathways and hub genes may represent novel therapeutic targets.

**Supplementary Information:**

The online version contains supplementary material available at 10.1007/s12975-020-00869-y.

## Introduction

Intracerebral hemorrhage (ICH) accounts for 10–15% of all strokes, and has high morbidity and mortality [1–4]. The hematoma causes mechanical brain injury [3] and disrupts the blood–brain barrier (BBB), resulting in edema formation and leukocyte extravasation from peripheral blood into damaged tissues [5, 6]. Perihematomal edema (PHE) results from transcapillary efflux of electrolytes and water from blood vessels. Additionally, osmotically active serum proteins along with neuronal energy failure result in a combination of vasogenic (BBB disruption) and cytotoxic edema (cell death) [7]. The inflammatory response, thrombin activation, coagulation factors, erythrocyte lysis, and various cytotoxic molecules contribute to PHE [7].

Roughly 30% of ICH risk is explained by genetic variants [8]. Hypertension, heavy alcohol consumption, cerebral amyloid angiopathy (CAA), smoking, low cholesterol, and some drugs increase ICH risk [9], yet much of ICH risk remains unexplained. Since gene expression combines genetic and environmental factors, it could contribute to understanding ICH risk. Moreover, since ICH and PHE volumes have a large effect on outcome [10, 11], the molecular changes associated with increasing ICH and PHE volumes might help discover novel treatment targets.

The immune system communicates with the central nervous system (CNS) through afferent and efferent cellular and molecular trafficking. Importantly, the peripheral immune system plays a major role in modulating damage and repair pathways following ICH [12–14]. Thus, we investigated the peripheral whole blood transcriptome to identify genes, co-expressed gene modules, and their hubs (possible master regulators) which correlate with ICH volume, absolute PHE volume (aPHE), and relative PHE (aPHE/ICH; rPHE).

## Methods

Detailed methods can be found in Online Resource 1.

### Study Subjects

The study protocol was approved by UC Davis Institutional Review Board and adheres to all federal and state regulations related to the protection of human research subjects, including The Common Rule, principles of The Belmont Report, and Institutional policies and procedures. Written informed consent was obtained from all participants or their proxy. In brief, peripheral venous blood was collected from ICH subjects and RNA isolated and prepared for hybridization on GeneChip^®^ Human Transcriptome Array (HTA) 2.0 as previously described [15]. Prior to analysis on Affymetrix HTA 2.0 arrays, RNA quality and purity were assessed on a Nanodrop ND-1000 spectrophotometer, and integrity was checked using the Agilent 2100 Bioanalyzer by calculating the ratio of 28S to 18S and the RIN number.

### Volumetric Measures

ICH volume and absolute PHE (aPHE) volume were measured on CT scans with AnalyzePro software. The ratio of aPHE to ICH volumes yielded the rPHE to adjust for ICH size [16].

### Per-Gene Analysis for Association with Volumetric Measures

Multiple regression models were performed in Partek Genomics Suite on log_2_ transformed data. Genes whose expression level correlated with ICH volume were modeled by *Y*_i_ = *μ* + ICHvol + Time_1_ + Time_2_ + ε_i_, where *Y*_i_ is the gene expression, *μ* is the common effect for the whole experiment, ICHvol is the ICH volume (cm^3^), Time_1_ is the time (hours) from symptom onset to blood draw, Time_2_ is the time (hours) from CT scan to blood draw, and ε_i_ is the random error. The aPHE volume and rPHE were modeled by *Y*_i_ = μ + aPHEvol + rPHE + Time_1_ + Time_2_ + ε_i_. A partial correlation between gene expression and the volumetric measures was calculated after accounting for the model’s covariates (*p* < 0.005 considered significant; partial correlations were with *r* > |0.6|).

### Weighted Gene Co-Expression Network Construction and Analysis

Data was imported into R where the function *goodSamplesGenes* within the Weighted Gene Co-Expression Network Analysis (WGCNA) package was used to identify missing values or zero-variance genes for removal from the dataset. WGCNA was run to identify networks, which are comprised of modules (groups of genes) with correlated/co-expressed genes using Pearson correlation [17]. An approximate scale-free topology was depicted by the data, as is expected of gene co-expression networks [18], and a soft-thresholding power (β) of 14 was chosen to maximize strong correlations between genes while minimizing weak correlations [18]. We used WGCNA’s signed network function to consider both positive and negative correlations [19]. Due to the large number of genes being processed, minimum module size was set at 100 genes. Rather than assigning a static cut-off during module construction, the *cutreeDynamic* function was used to form modules due to its adaptability to complex dendrograms that allows for identifying nested modules [20]. Hub genes were defined as the top 5% based on their interconnectivity (kIN—the gene’s intramodular connectivity) [21, 22].

### Correlation of Volumetric Parameters with the WGCNA Modules

WGCNA correlated the volumetric parameters and other technical, clinical, and demographic parameters with each module’s eigengene to identify modules which have significant correlation. Correlations with ICH volume, aPHE volume, rPHE size, and/or other parameters were considered significant with *p* < 0.05.

### Cell-Specific Gene Involvement

To identify enrichment with blood cell type-specific genes, the significant gene lists were overlapped with lists of blood cell type-specific genes [23, 24]. The overlaps’ significance was assessed using hypergeometric probability testing (R function *phyper*; *p* < 0.05 considered significant).

### Pathway and Gene Ontology Analyses

Ingenuity Pathway Analysis (IPA^®^, QIAGEN) was performed on all gene lists as previously described [25]. Pathways with Benjamini-Hochberg (BH) multiple-comparison adjusted *p* < 0.05 were considered significant. IPA’s pathway activity prediction analysis was used to determine if the significant pathways were activated or inhibited based on the expression direction (correlation coefficient) of the input genes. IPA’s Z-score algorithm calculated the predicted overall activation/inhibition states of the canonical pathways by statistically comparing our uploaded datasets with the IPA knowledge base’s expression patterns [26]. For the per-gene analysis, we used the partial correlation coefficients associated with each gene from each of the gene lists. For the network analyses’ results, however, since WGCNA calculates the correlation between the volumetric parameter and module’s eigengene, and since the co-expressed genes within each module might have different sign correlations, we calculated the Pearson correlation between each gene from the significant WGCNA modules and the particular volumetric measure. This correlation value was input into IPA for prediction of pathways’ activation/suppression status. IPA calculates the canonical pathway’s prediction by considering the activation state of one or more key molecules when the Pathway is activated, as well as the molecules’ causal relationships with each other. It generates an activity pattern for the molecules and the end-point functions in the pathway. Canonical pathways with *Z* ≥ 2 are considered significantly activated, while ones with *Z* ≤ −2 are considered significantly suppressed. Since we input correlation coefficients and not fold-changes of differential expression between disease and control, pathways with significant activation mean that the higher the volumetric measure (ICH volume, aPHE volume, or rPHE), the more activated the pathways. Similarly, for a pathway with significant suppression, it would mean that the higher the volumetric measure, the more suppressed the pathway.

DAVID Functional Annotation Bioinformatics Resources Database was used to identify enrichment with relevant biological processes (EASE-adjusted False Discovery Rate (FDR) *p* value < 0.05) [27, 28].

## Results

### Demographic and Clinical Characteristics

Demographic and clinical characteristics are presented in Table 1. ICH and aPHE volumes and rPHE size were 21 ± 22 cm^3^ (Mean ± SD), 31 ± 34 cm^3^, and 1.8 ± 1.3 respectively (Table 1). Thirty-day survival was available for 9/18 subjects, of which 2/9 died.

**Table 1:** Demographic and clinical characteristics

ICH patient demographics	
Subjects (#)	18
Sex (M, F)	13, 5
Age (years; Mean ± SD)	61.4 ± 13.9
Min, Max	37, 83.85
Q1, Q2, Q3	50.32, 62.50, 71.69
Diabetes (#)	2
Hypercholesterolemia (#)	5
Hypertension (#)	13
Smoking status (#)	
Current smoker	2
Ex-smoker	5
Non-smoker	10
Unknown	1
Race (#)	
Black	4
Latino	4
Asian	2
White	6
Mixed	2
Time from onset to blood draw (h; Mean ± SD)	61.7 ± 33.9
Min, Max	4.23, 124.25
Q1, Q2, Q3	32.35, 65.33, 87.77
Time from CT Scan to blood draw (h; Mean ± SD)	40.8 ± 28.7
Min, Max	3.08, 93.17
Q1, Q2, Q3	20.48, 30.45, 61.97
Time from onset to CT scan (h; Mean ± SD)	20.9 ± 31.1
Min, Max	1.15, 102.78
Q1, Q2, Q3	2.83, 9.06, 17.47
ICH location (#)	
Cortical	7
Deep	11
ICH cause (#)	
Hypertensive	11
Amyloid angiopathy	3
Unknown (cortical, likely CAA)	4
ICH volume (cm^3^; Mean ± SD)	20.94 ± 21.78
Min, Max	0.04, 83.8
Q1, Q2, Q3	5.24, 10.46, 30.76
Perihematomal edema volume (aPHE) (cm^3^; Mean ± SD)	31.30 ± 33.75
Min, Max	2.60, 117.76
Q1, Q2, Q3	8.90, 14.79, 49.28
Relative PHE size (rPHE) (Mean ± SD)	1.83 ± 1.25
Min, Max	0.66, 6.51
Q1, Q2, Q3	1.33, 1.47, 1.88

### Per-Gene Correlation Analysis

#### Genes Associated with ICH Volume

**Fig. 1:**
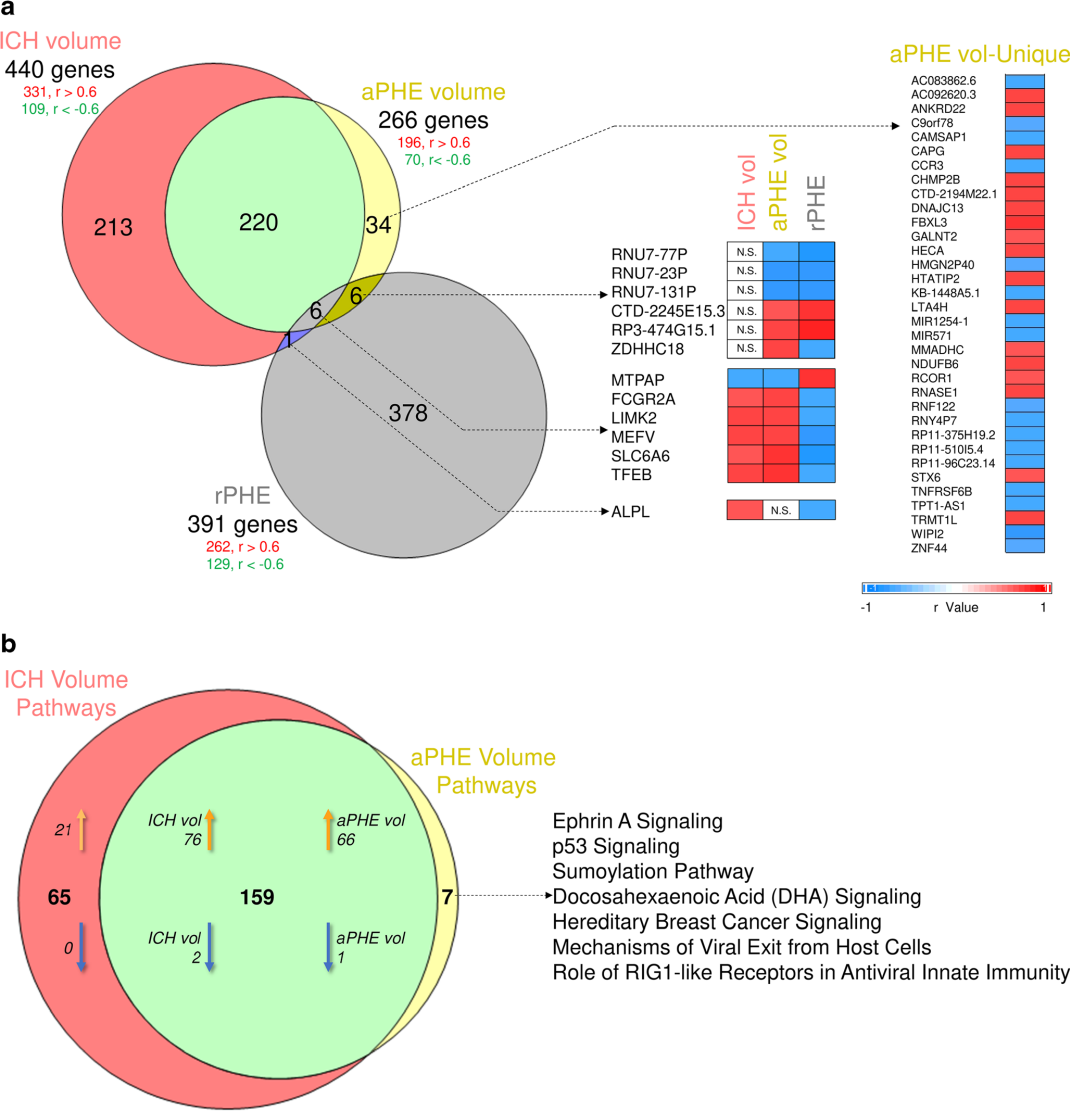
Overlap between genes correlating with the volumetric measures in the per-gene analyses (**a**) and the overrepresented pathways (**b**). All non-significant *r* values are displayed as white cells, labeled N.S. (non-significant). In (**b**), orange arrows denote the number of predicted activated pathways, while blue arrows denote the number of predicted suppressed pathways

**Fig. 2:**
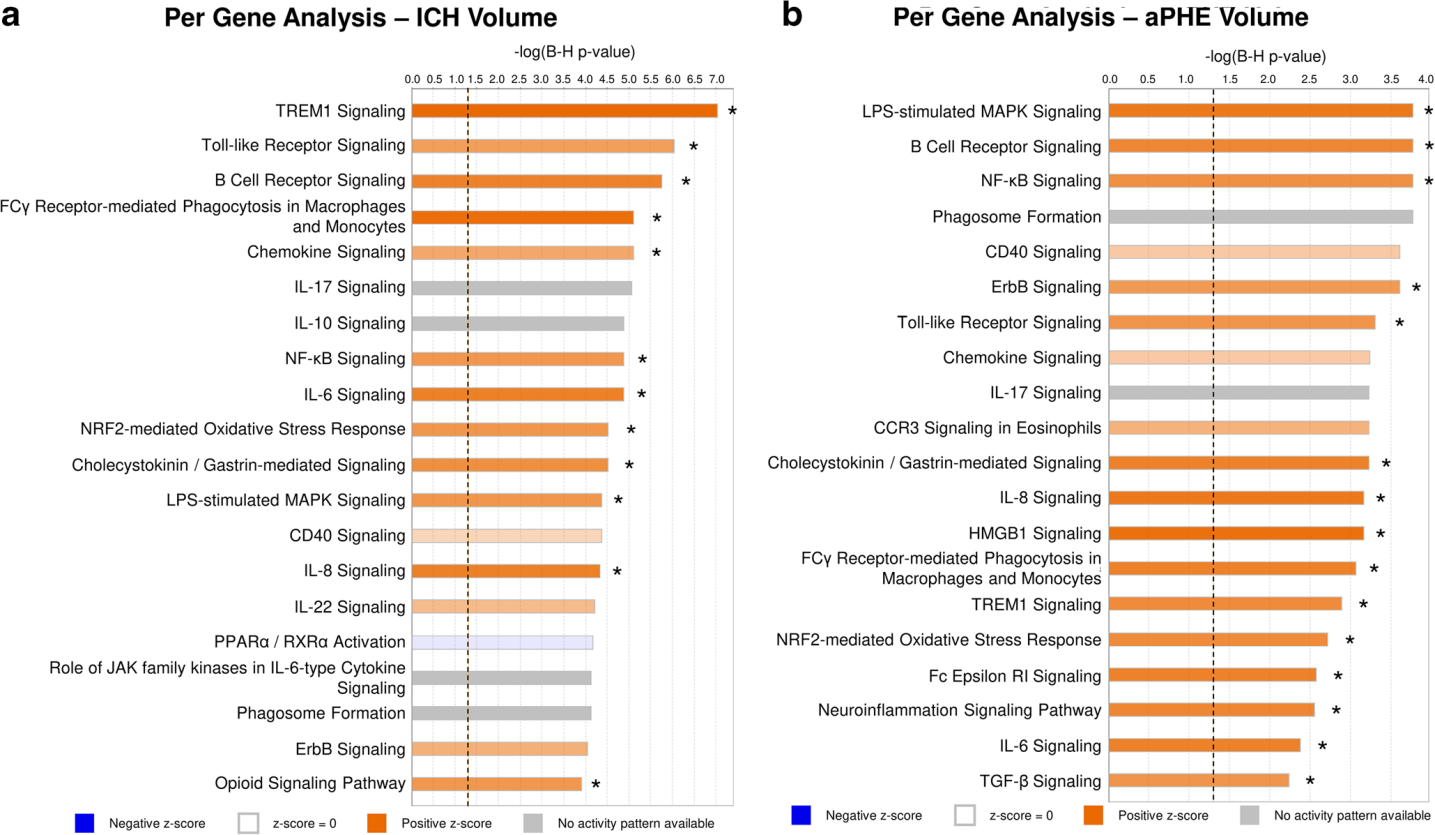
Top 20 relevant overrepresented pathways in ICH volume per-gene analysis (**a**), aPHE volume per-gene analysis (**b**). Asterisk denotes significant activation (*Z* ≥ 2) or suppression (*Z* ≤ − 2)

Expression of 440 genes correlated significantly with ICH volume: 109 negatively, and 331 positively (Fig. 1a, Table S1A). Among them were precursor miRNAs and lincRNA (long intergenic non-coding RNA). The 440 genes were enriched in 224 pathways (Fig. 1b), of which 97 were activated while two (PPAR and Apoptosis Signaling pathways) were significantly suppressed with increased ICH volume (Fig. 2a, Table S2A). Activated pathways included Neuroinflammation (GSK3B, ICAM1, IFNGR), NF-kB, Toll-Like Receptor (TLR) signaling, and NRF2-Mediated Oxidative Stress Response. Gene Ontology (GO) analysis included Regulation of Transcription Factor Binding Activity and Inflammatory Response with genes such as Toll-like receptors (TLR1, 5, 8, 10) and NF-kB inhibitor zeta (NFKBIZ) (FDR *p* < 0.05; Table S3A). Additionally, 65/440 genes (15%) are reported to be granulocyte-specific (primarily neutrophils; *p*(overlap) < 1e-16), including GSK3B and CAMK2G [29–33] (Table S4A).

#### Genes Associated with aPHE Volume

Expression of 266 genes correlated with aPHE volume: 70 negatively and 196 positively (Fig. 1a, Table S1B). Among them were several precursor miRNAs. The 266 genes were over-expressed in 166 pathways (Fig. 1b), with 66 activated and one suppressed (PPAR Signaling; Fig. 2b, Table S2B). Activated pathways included TLR, NF-kB, HMGB1, TREM1, and Neuroinflammation Signaling. Cellular Response to Mechanical Stimulus and Protein Metabolic processes were overrepresented GO terms, including SLC38A2 (aka SNAT2, ATA2, SAT2; Table S3B). Additionally, 41/266 genes (15%) are reported neutrophil-specific (*p*(overlap) = 5.4e-14). There was substantial overlap between many aPHE and ICH volume genes (Table S4B).

#### Genes Associated with rPHE

Expression of 391 genes correlated significantly with rPHE: 129 negatively and 262 positively (Fig. 1a, Table S1C). Among them were 15 precursor miRNAs; six small nucleolar RNA (snoRNA), typically involved in mRNA splicing; and four lincRNA. No pathway passed Benjamini-Hochberg-corrected *p* < 0.05; 12 pathways passed uncorrected *p* < 0.05 (Table S2C). Gene Ontology identified significant enrichment (FDR *p* < 0.05) in the JNK Cascade and Mitochondrial Translation Elongation, including 7 nuclear genes encoding 5 mitochondrial ribosomal proteins, mitochondrial translation elongation factor, and mitochondrial small ribosomal subunit protein (Table S3C). Additionally, 31/391 genes (8%) that associated with rPHE have been reported neutrophil-specific (*p*(overlap) = 1.7e-04), including AQP9 (aquaporin 9), TREM1, and TNFRSF10C (Table S4C).

### Weighted Gene Co-Expression Network Analysis

#### Co-Expressed Gene Modules Correlated with ICH Volume, aPHE Volume, and rPHE

**Fig. 3:**
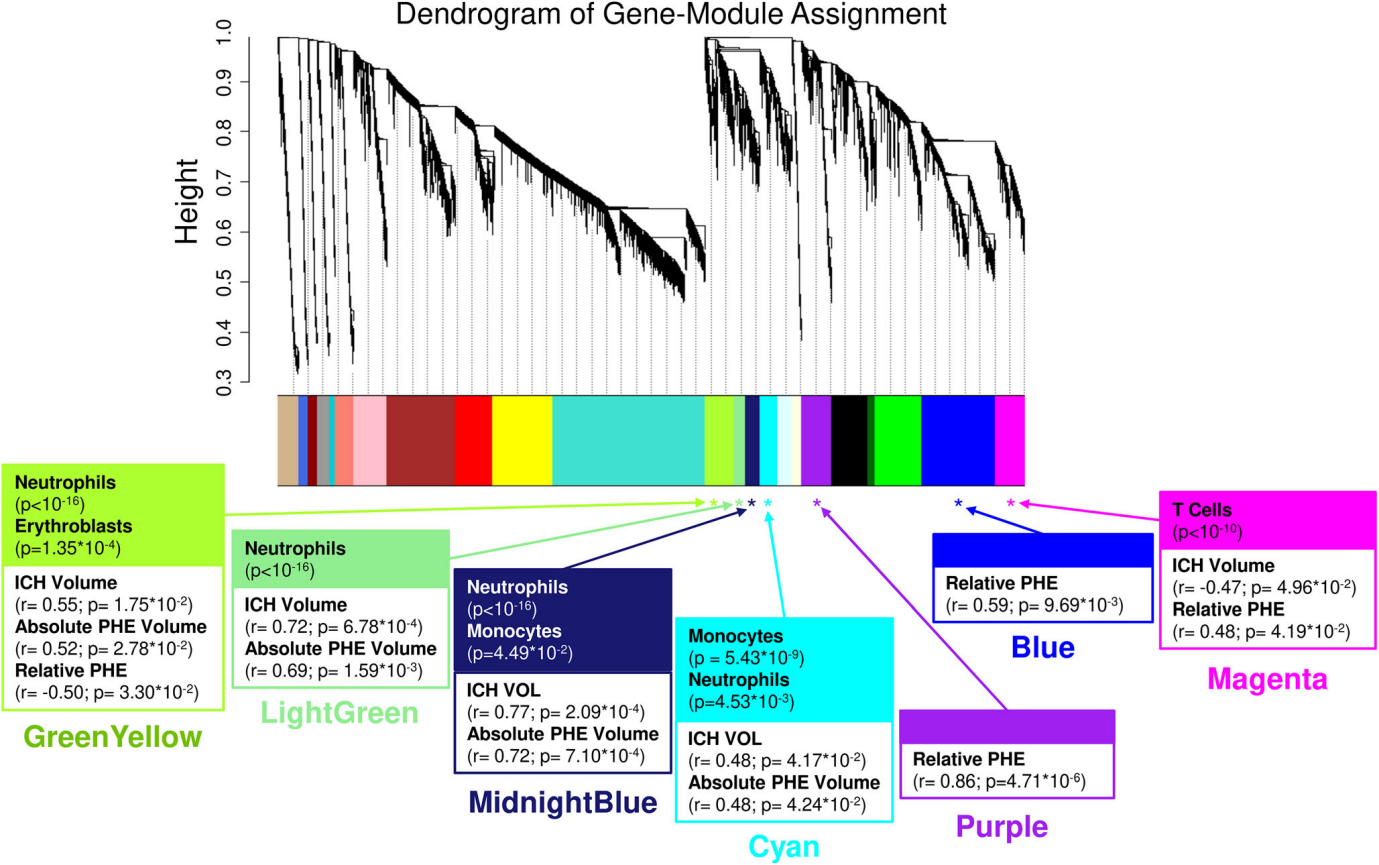
Dendrogram of all analyzed genes, clustered in WGCNA-identified co-expressed modules. The seven modules significant to the volumetric parameters are indicated as well as their cell type enrichments

**Fig. 4:**
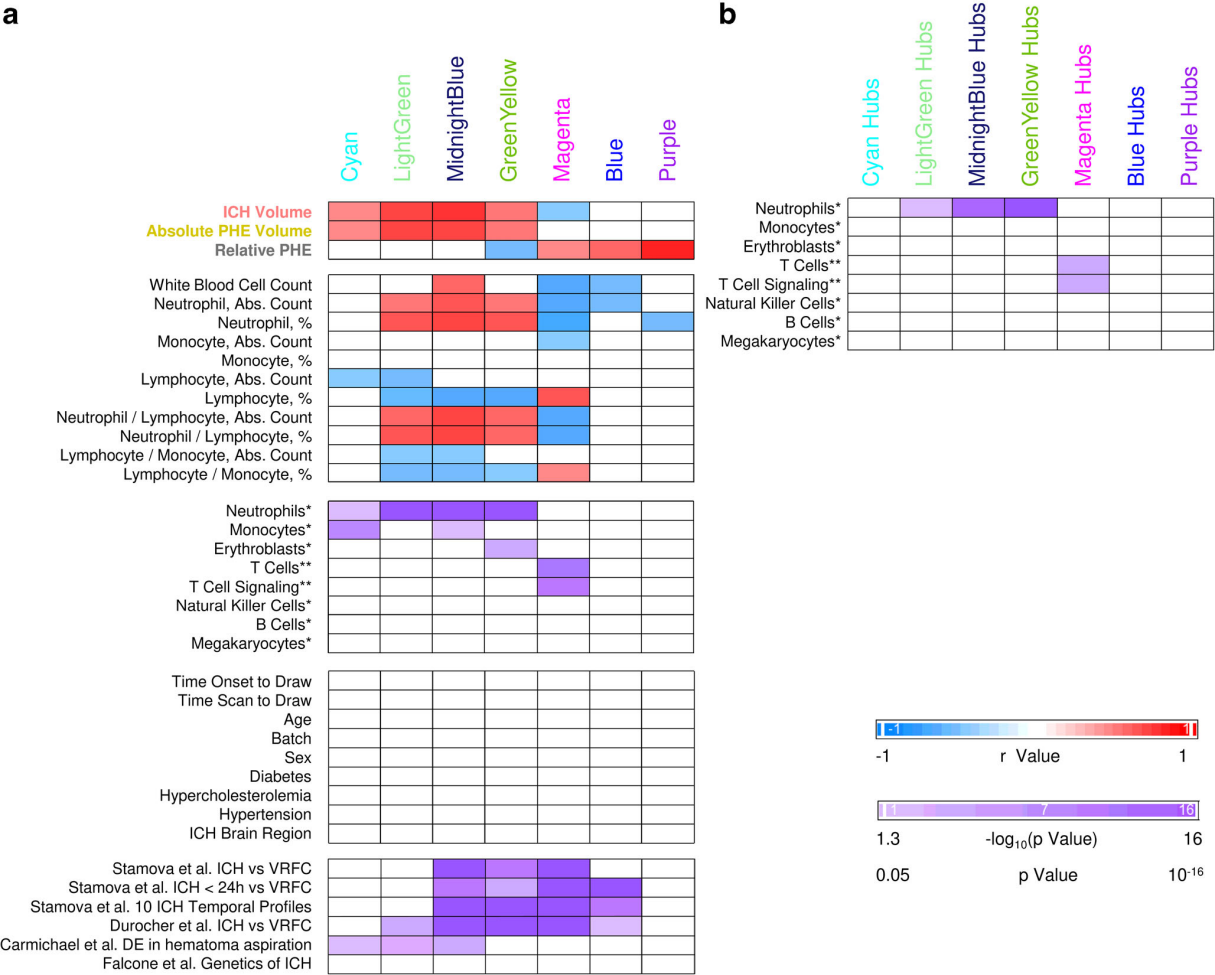
Association with clinical parameters and cell-specific gene enrichment in the seven significant modules (**a**) and their hubs (**b**). All non-significant *r* values or non-significant hypergeometric probabilities of overlaps are displayed as white cells. *Watkins et al. [23]. **Chtanova et al. [24]. Please note, for more comprehensive coverage of T cell-specific genes we overlapped our findings with ST1 and ST2 from Chtanova et al. [24]: ST 1: genes selectively expressed in T cells, ST2: genes selectively expressed in T cells and involved in the TCR complex, co-stimulation and signaling. No significant overlap with the Watkins Th and Tc lists were found. Stamova et al. [15] differential expression was at individual transcript-isoform level, while Durocher et al. [17] was at gene level. Overlap with these studies was performed by gene symbols. *DE* - differentially expressed

**Fig. 5:**
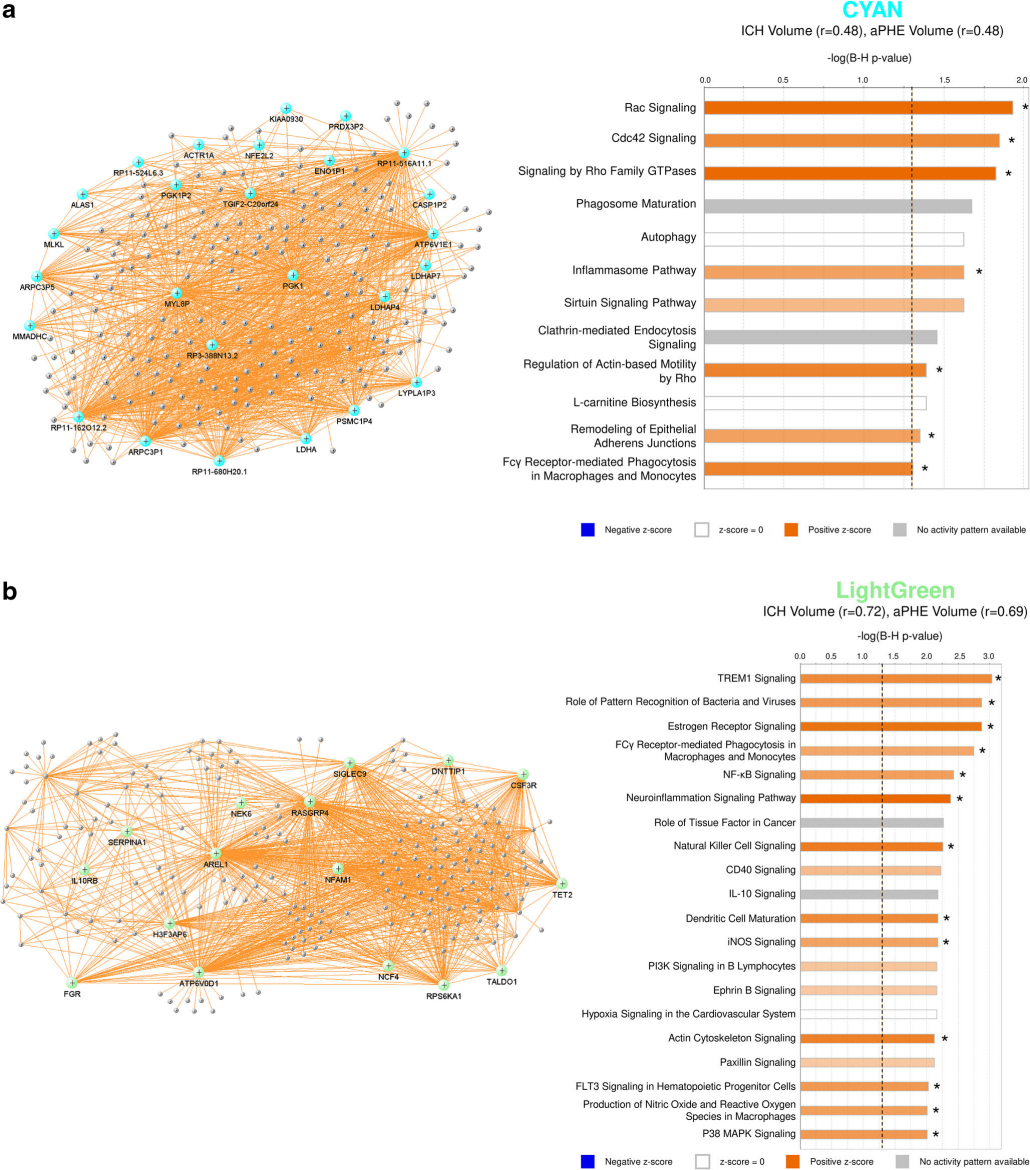

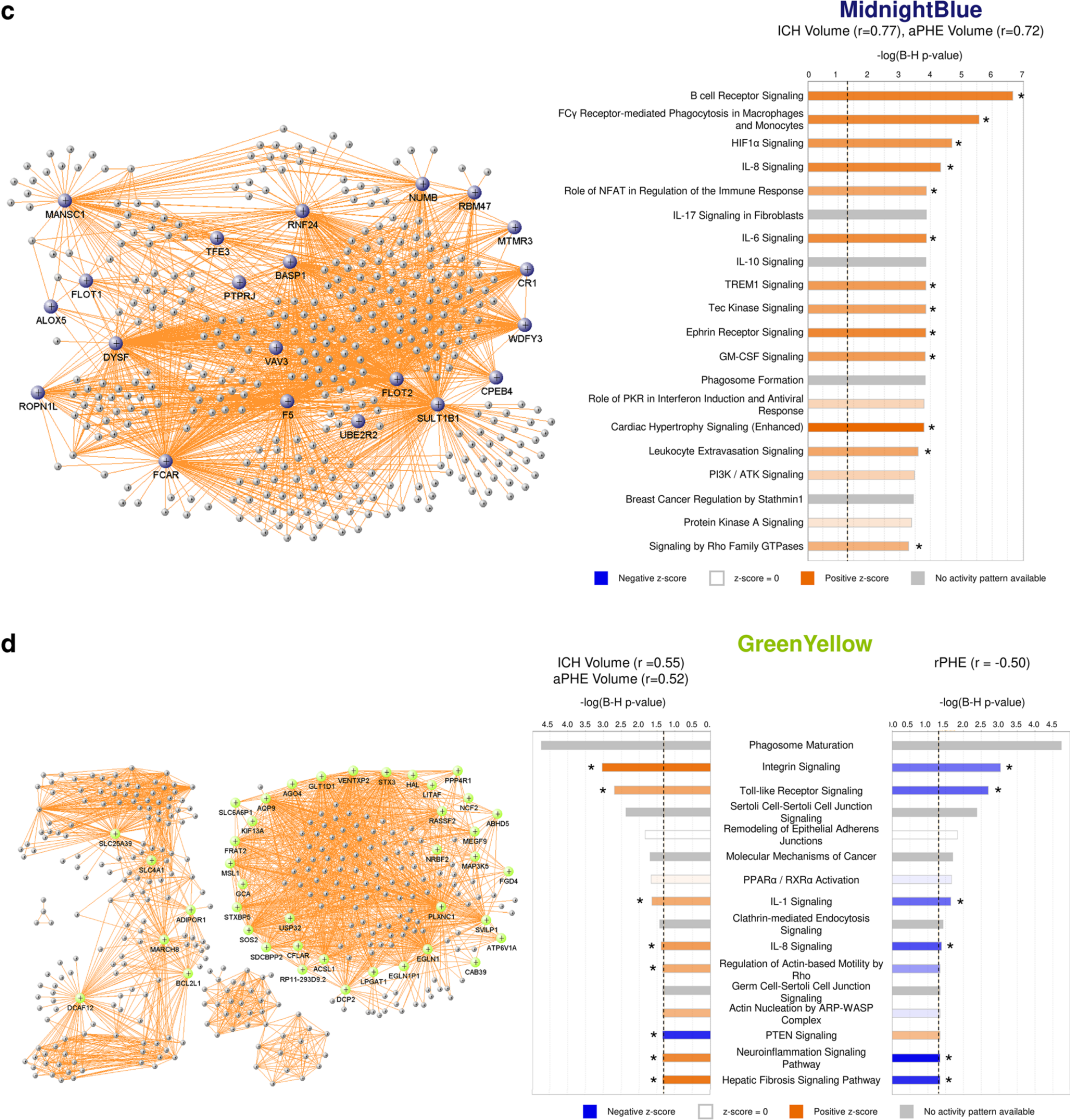

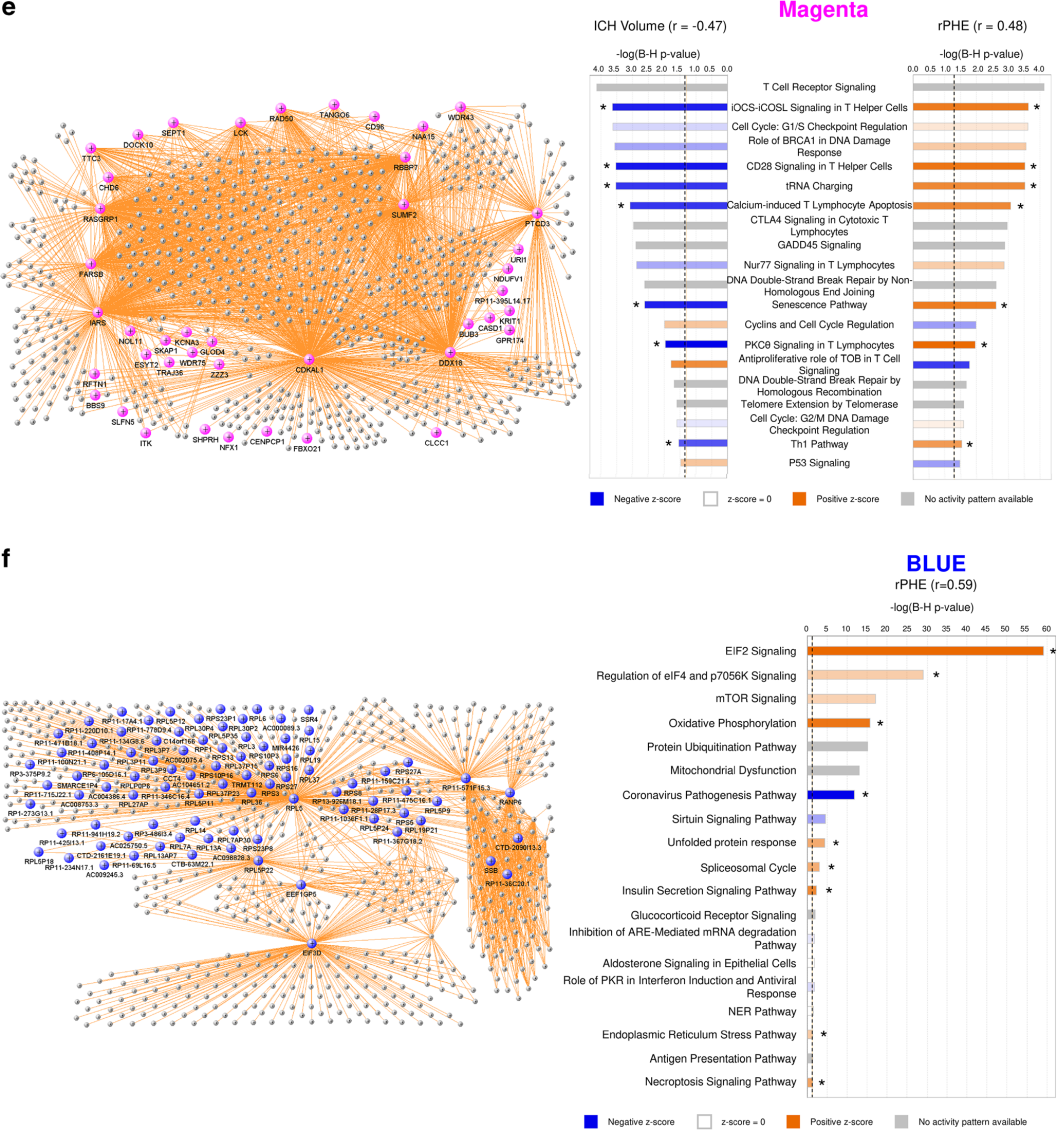

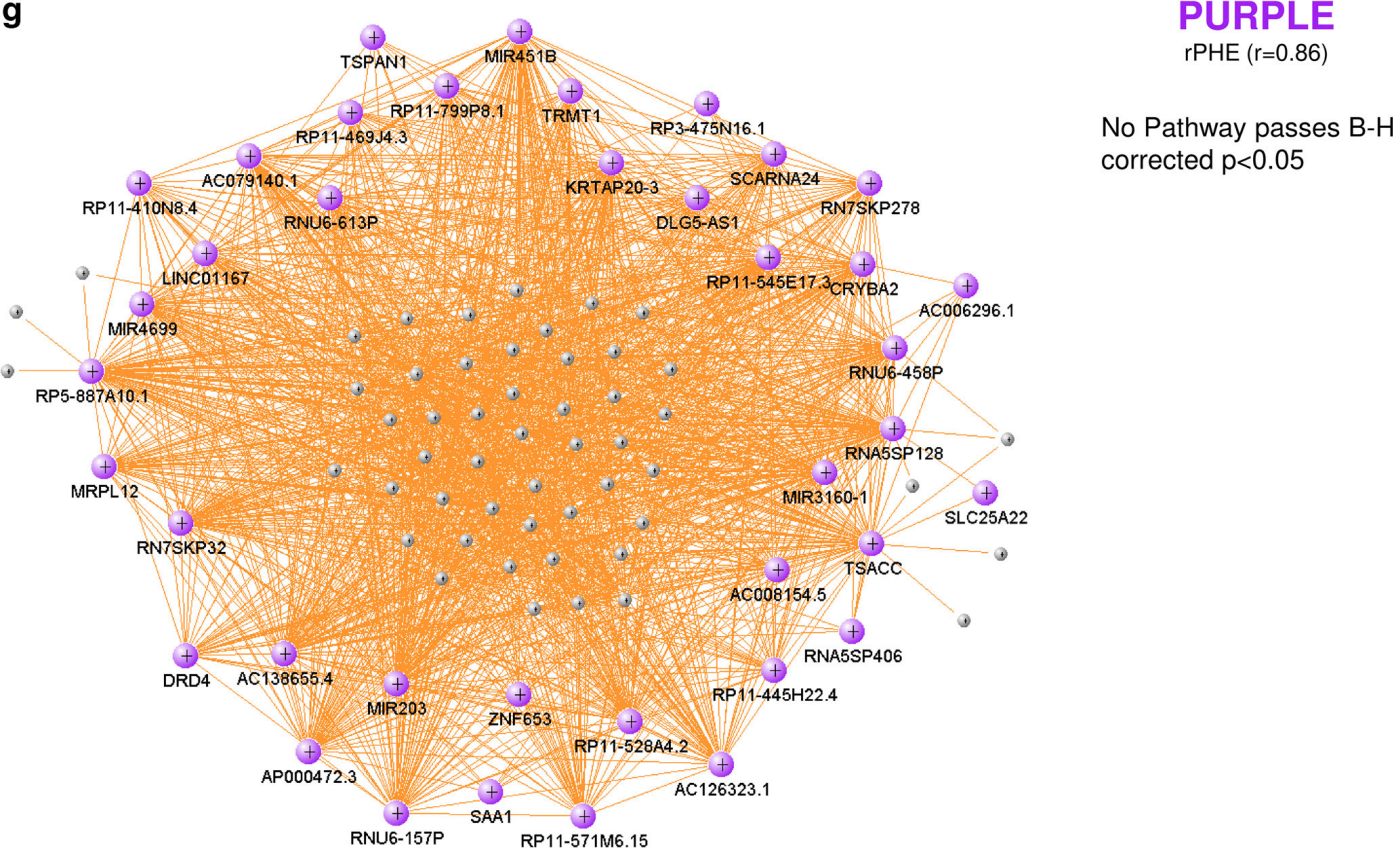
VisANT networks (left panels) and IPA analyses (right panels) of the genes in the Cyan (**a**), LightGreen (**b**), MidnightBlue (**c**), GreenYellow (**d**), Magenta (**e**), Blue (**f**), and Purple (**g**) modules. The colored genes in the left panels are hub genes. Asterisks in the right panels represent significantly activated (*Z* ≥ 2) or suppressed (*Z* ≤ − 2) pathways. The GreenYellow module (**d**) was positively associated with the ICH volume, the aPHE volume, and negatively with rPHE. Of the positive associations, in (**d**) we present only the IPA results for the ICH volume. The aPHE results were similar, with the exception of IL-8 Signaling showing a trend towards activation (*Z* = 1.94) and PTEN Signaling not significantly suppressed (*Z* = − 0.91)

Twenty-three modules of co-expressed genes and a single module (Gray) of non-co-expressed genes were identified. Figure 3 shows a dendrogram of all 21,175 genes and their respective module assignments. Modules were tested for significance to each parameter (Fig. 4a). Seven modules showed significant association to the volumetric measures: five to ICH volume (Cyan, GreenYellow, LightGreen, Magenta, and MidnightBlue); four to aPHE volume (Cyan, GreenYellow, LightGreen, and MidnightBlue); and four to rPHE (GreenYellow, Magenta, Blue, and Purple) (Fig. 4a; Table S5,S6). All modules showed positive correlation between the eigengene and the volumetric measures, except Magenta and GreenYellow (negatively correlating with ICH volume and rPHE, respectively; Fig. 4a). Table S6A-X show values calculated for each module for each trait. Modules’ networks are presented in Fig. 5 (left panels) along with their hubs in both Table 2 and the colored nodes in Fig. 5. Notably, 74/103 (72%) of Blue’s hubs were ribosomal proteins (Table 2).

**Table 2:** Hub genes in the modules significant to the volumetric measures

Cyan	LightGreen	MidnightBlue	GreenYellow	Magenta	Blue			Purple
ACTR1A	AREL1	ALOX5	ABHD5	BBS9	AC000089.3	RP11-36C20.1	RPL4P5	AC006296.1
ALAS1	ATP6V0D1	BASP1	ACSL1	BUB3	AC002075.4	RP11-408P14.1	RPL5	AC008154.5
ARPC3P1	CSF3R	CPEB4	ADIPOR1	CASD1	AC004386.4	RP11-425I13.1	RPL5P11	AC079140.1
ARPC3P5	DNTTIP1	CR1	AGO4	CD96	AC008753.3	RP11-471B18.1	RPL5P12	AC126323.1
ATP6V1E1	FGR	DYSF	AQP9	CDKAL1	AC009245.3	RP11-475C16.1	RPL5P18	AC138655.4
CASP1P2	H3F3AP6	F5	ATP6V1A	CENPCP1	AC016739.2	RP11-571F15.3	RPL5P22	AP000472.3
ENO1P1	IL10RB	FCAR	BCL2L1	CHD6	AC025750.5	RP11-69L16.5	RPL5P24	CRYBA2
KIAA0930	NCF4	FLOT1	CAB39	CLCC1	AC098828.3	RP11-715J22.1	RPL5P34	DLG5-AS1
LDHA	NEK6	FLOT2	CFLAR	DDX18	AC104651.2	RP11-778D9.4	RPL5P35	DRD4
LDHAP4	NFAM1	MANSC1	DCAF12	DOCK10	C14orf166	RP11-941H19.2	RPL5P9	GLI4
LDHAP7	RASGRP4	MTMR3	DCP2	ESYT2	CCT4	RP1-273G13.1	RPL6	KRTAP20-3
LYPLA1P3	RPS6KA1	NUMB	EGLN1	FARSB	CTB-63M22.1	RP13-926M18.1	RPL7A	LINC01167
MLKL	SERPINA1	PTPRJ	EGLN1P1	FBXO21	CTD-2090I13.3	RP3-375P9.2	RPL7AP30	MIR203^a^
MMADHC	SIGLEC9	RBM47	FGD4	GLOD4	CTD-2161E19.1	RP3-486I3.4	RPLP0P6	MIR3160-1^a^
MYL8P	TALDO1	RNF24	FRAT2	GPR174	EEF1G	RP6-105D16.1	RPS10P16	MIR451B^a^
NFE2L2 (aka NRF2)	TET2	ROPN1L	GCA	IARS	EEF1GP5	RPF1	RPS10P3	MIR4699^a^
PGK1		SULT1B1	GLT1D1	ITK	EIF3D	RPL13A	RPS13	MRPL12
PGK1P2		TFE3	HAL	KCNA3	EIF3M	RPL13AP7	RPS16	NSG1
PRDX3P2		UBE2R2	KIF13A	KRIT1	MINOS1	RPL14	RPS23P1	RN7SKP278
PSMC1P4		VAV3	LITAF	LCK	MIR4426^a^	RPL15	RPS23P8	RN7SKP32
RP11-162O12.2		WDFY3	LPGAT1	NAA15	NDUFB8P2	RPL19	RPS27	RNA5SP128
RP11-516A11.1			MAP3K5	NDUFV1	RANP6	RPL19P21	RPS27A	RNA5SP406
RP11-524 L6.3			MARCH8	NFX1	RP11-100N21.1	RPL27AP	RPS3	RNU6-157P
RP11-680H20.1			MEGF9	NOL11	RP11-1036F1.1	RPL3	RPS5	RNU6-458P
RP3-388 N13.2			MSL1	PTCD3	RP11-1072N2.2	RPL30P2	RPS6	RNU6-613P
TGIF2-C20orf24			NCF2	RAD50	RP11-118D22.3	RPL30P4	RPS8	RP11-410N8.4
			NRBF2	RASGRP1	RP11-134G8.6	RPL36	RSL1D1	RP11-445H22.4
			PLXNC1	RBBP7	RP11-142L4.3	RPL37	SMARCE1	RP11-469J4.3
			PPP4R1	RFTN1	RP11-159C21.4	RPL37P15	SMARCE1P4	RP11-528A4.2
			RASSF2	RP11-395L14.17	RP11-17A4.1	RPL37P23	SSB	RP11-545E17.3
			RP11-293D9.2	SEPT1	RP11-220D10.1	RPL3P11	SSR4	RP11-571M6.15
			SDCBPP2	SHPRH	RP11-234N17.1	RPL3P7	TRMT112	RP11-799P8.1
			SLC25A39	SKAP1	RP11-28P17.3	RPL3P9	UBA2	RP11-97E7.2
			SLC4A1	SLFN5	RP11-346C16.4	RPL4		RP3-475N16.1
			SLC6A6P1	SUMF2	RP11-367G18.2	RPL4P4		RP5-887A10.1
			SOS2	TANGO6				SAA1
			STX3	TRAJ36				
			STXBP5	TTC3				
			SVILP1	URI1				
			USP32	WDR43				
			VENTXP2	WDR75				
				ZZZ3				

^a^MIR gene names represent precursor (immature) microRNAs

#### Cell Type Involvement in Volumetric Measure-Correlating Modules

Hypergeometric probability testing on the seven significant modules associated with volumetric measures was conducted against genes specifically expressed in individual blood cell types (Fig. 4a) [23, 24]. Chtanova et al. was used for T cell-specific gene representation [24]. Four modules were enriched in granulocyte-specific (mainly neutrophil) genes (Cyan, GreenYellow, LightGreen, and MidnightBlue). MidnightBlue and Cyan were also enriched in monocyte-specific genes, GreenYellow in erythroblast-specific genes, and Magenta in T cell-receptor and other T cell genes [24](Fig. 4a, Table S4D-J).

To identify which cell types are driving the correlation with the volumetric measures, we performed hub-gene cell enrichment analysis. GreenYellow, LightGreen, and MidnightBlue hubs were enriched in neutrophil-specific genes, and Magenta hubs in T cell receptor and other T cell specific genes (Fig. 4b, Table S4K-Q).

#### Clinical and Demographic Characteristics in Modules Correlated to Volumetric Measures

Several modules were associated with blood cell count, lymphocytes, neutrophils, monocytes, and ratio of neutrophils-to-lymphocytes and/or lymphocytes-to-monocytes (Fig. 4a). There was no significant association with sex, age, vascular risk factors, time, and batch (Fig. 4a, Table S6D-X).

#### Pathways in Modules and Hubs Associated with Volumetric Measures

**Fig. 6:**
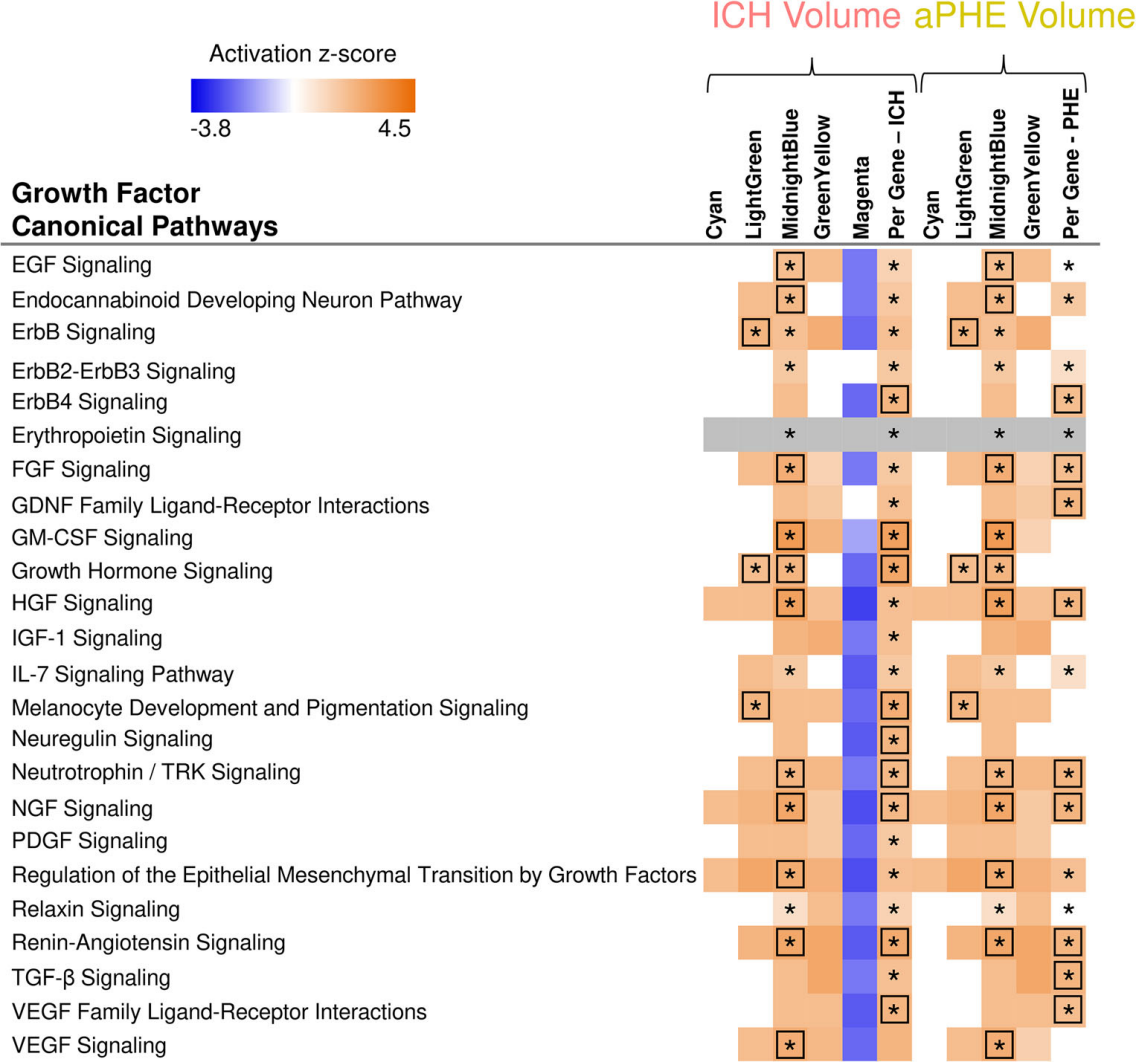
Growth factor canonical pathway enrichment for per gene and module lists. An asterisk denotes BH significant enrichment in a pathway (BH *p* < 0.05); among those—a box denotes significant activation or suppression in pathways that are significantly enriched (|*Z*| ≥ 2). Shading represents predicted pathway activation (orange) and suppression (blue)

Cyan correlated with ICH and aPHE volumes and had 12 significant pathways such as inflammatory and autophagy pathways (including 7 activated with larger volumes; Fig. 5a, right panels; Table S2D,E). LightGreen had 64 significant pathways such as Hypoxia Signaling (including HIF-1α [34]). Of those, 36 were activated including Apelin Cardiomyocyte Signaling, NF-kB, TLR, Pattern Recognition Receptors, Growth Hormone Signaling, and LPS-induced MAPK Signaling pathways (Fig. 5b, right panel; Fig. 6; Table S2F,G). MidnightBlue had 189 significant pathways that correlated with ICH and aPHE volumes, including 103 activated and two suppressed (PPAR Signaling and Antioxidant Action of Vitamin C) (Fig. 5c; Table S2H,I). GreenYellow, positively correlated with ICH and aPHE volumes and negatively with rPHE, had 16 significant pathways: 9 activated and one (PTEN Signaling) suppressed in subjects with larger ICH volumes; 6 suppressed in rPHE (Fig. 5d, Table S2J-L). Magenta, negatively correlated with ICH volume and positively with rPHE, had 33 significant pathways: 13 were suppressed with increasing ICH volumes including PKCθ Signaling in T Lymphocytes, CD28 Signaling in T Helper Cells, iCOS-iCOSL Signaling in T Helper Cells, Calcium-Induced T Lymphocyte Apoptosis, and Th1 Pathway. The same 13 pathways were activated with increasing rPHE (Fig. 5e; Table S2M,N). Blue positively correlated with rPHE and had 20 pathways including 8 activated (including EIF signaling, eIF4 and p70S6K Signaling, stress response pathways (UPR, ER) and Necroptosis Signaling; Fig. 5f, Table S2O). Purple did not have any significant pathways (Fig. 5g). Hub gene pathways are presented in Table S2P-R.

Gene Ontology (GO) enrichment analysis (Table 3, Table S3) showed Magenta and Blue were dominated by RNA splicing, translation, and protein processing pathways and T cell receptor signaling. Autophagy/autophagosome assembly populated Cyan and GreenYellow; apoptotic processes in LightGreen and MidnightBlue; platelet activation/degranulation in GreenYellow and MidnightBlue; immune/inflammatory responses in LightGreen, GreenYellow, Cyan, and MidnightBlue.

**Table 3:** Top GO biological process terms and their genes in the significant modules

Module	GO Term	Genes
Cyan	Autophagy (FDR *p* = 1.0E-04)	BECN1, CHMP3, EPG5, HGS, IFI16, LAMP1, MAP1LC3B, RAB24, RGS19, S100A8, TBC1D5, VCP, VTA1
	Cell-cell adhesion (FDR *p* = 1.8E-04)	ABI1, CAPZA1, CRKL, DBNL, ELMO2, EPS15L1, HIST1H3E, HIST1H3G, HSPA5, IST1, LDHA, LRRFIP1, PKM, RTN4, SCYL1, SNX1, STK24, USP8
	Innate immune response (FDR *p* = 1.7E-02)	ADAR, ANXA1, APOBEC3B, APP, BTK, CAPZA1, CAPZA2, CD300E, CLEC4A, CYBB, FCER1G, FES, HAVCR2, IFI16, MX2, NFKB1, S100A8, TIRAP, UBC
LightGreen	Signal transduction (FDR *p* = 2.4E-05)	ARAP3, ARHGAP9, ARRB2, ATF6, AVP, CAP1, CDC42SE1, CREBBP, CSF3R, CSNK1D, FCGR2B, FYB, GNAT1, HIF1A, IGF1R, IL10RB, IL1B, IRAK4, LILRA2, LILRA3, NEK6, NFAM1, NLRP3, OSTF1, PIK3CD, PPP2R5A, RASA2, RPS6KA1, S100A6, SIRPB1, TANK, TLE3, TNFAIP6, TNFSF10, TNFSF13B, TNFSF14, TSPO
	Apoptotic process (FDR *p* = 1.1E-03)	AREL1, AVP, BNIP2, CARD8, CD14, HTATIP2, IL1B, MAPK3, NEK6, NLRP3, NUAK2, PAK1, PIM3, RPS6KA1, RRAGC, SRA1, TNFSF10, TNFSF14, TSPO, UBE2D3, ZFP36L1
	Immune response (FDR *p* = 2.6E-03)	CST7, FCGR2B, FYB, IGF1R, IL10RB, IL1B, IL1RN, ITGAD, MBP, NCF4, NOTCH1, PGLYRP1, SLPI, TNFSF10, TNFSF13B, TNFSF14, VAV1
MidnightBlue	Inflammatory response (FDR *p* = 4.0E-06)	ADAM8, BCL6, C5AR1, CASP4, CEBPB, CXCR1, CXCR2, FPR1, HCK, LYN, MEFV, MMP25, NAIP, NFKBIZ, NLRC4, PIK3CG, PROK2, PXK, SLC11A1, TLR5, TLR8, TNFRSF10D, TNFRSF1A, TNFRSF9, VNN1
	Platelet activation (FDR *p* = 5.5E-03)	ACTN1, F5, GNA13, GNAQ, LYN, MAPK1, PIK3CG, PLSCR1, PRKCD, VAV3
	Apoptotic process (FDR *p* = 1.9E-02)	C5AR1, CASP4, CASP5, GADD45A, HIPK3, KIF1B, MAP2K6, MAPK1, MAPK14, MEF2A, NAIP, NFKBIA, NLRC4, NOTCH2, PLSCR1, PRKCD, PTK2B, RALB, RNF144B, RTN3, SH3GLB1, STK3, TNFRSF9
GreenYellow	Autophagosome assembly (FDR *p* = 4.6E-04)	ATG16L2, GABARAPL1, GABARAPL2, RAB1A, STX12, TP53INP1, WDR45, WIPI1, WIPI2
	Platelet degranulation (FDR *p* = 4.9E-03)	CD36, CD9, CLU, F13A1, ITGA2B, ITGB3, LAMP2, PF4, PPBP, SELP, SPARC, VCL
	Innate immune response (FDR *p* = 5.0E-03)	AIM2, APOBEC3A, BMX, CAMP, CLEC4E, CLU, DDX3X, DEFA3, DEFA4, DEFA5, FBXO9, HLA-B, JAK2, LCN2, LGALS3, LY96, MAP3K5, NCF1, NCF2, PCBP2, TLR1, TLR10, TLR4, TOLLIP, TREM1, TREML1, UBA52, UBB
Magenta	mRNA splicing, via spliceosome (FDR *p* = 3.4E-08)	CSTF3, DBR1, FIP1L1, GEMIN5, HNRNPA1, HNRNPH1, HNRNPL, HNRNPR, METTL3, PDCD7, POLR2L, PPIH, PPWD1, PRPF19, PRPF4, PRPF4B, PRPF6, RBM41, RBMX, SF3A2, SF3A3, SF3B3, SKIV2L2, SNRPB, SNRPN, SNURF, SRSF10, SRSF7, SYNCRIP, TRA2B
	T Cell receptor signaling pathway (FDR p = 1.1E-04)	CARD11, CD247, CD3E, CD3G, CUL1, GATA3, GRAP2, ITK, LAT, LCK, MALT1, PIK3R1, PLCG1, PRKCQ, RFTN1, SKAP1, THEMIS, TRAC, ZAP70
	Protein sumoylation (FDR *p* = 3.5E-04)	AAAS, NDC1, NSMCE4A, NUP155, NUP160, NUP205, NUP43, NUP85, NUP93, PARP1, SCMH1, SMC5, SMC6, TOP2B, TP53, ZNF451
Blue	Translational initiation (FDR *p* = 1.6E-69)	ABCE1, DHX29, EIF1AX, EIF2A, EIF2B2, EIF2S1, EIF2S2, EIF2S3, EIF3A, EIF3C, EIF3D, EIF3E, EIF3G, EIF3H, EIF3I, EIF3J, EIF3K, EIF3L, EIF3M, EIF4A1, EIF4A2, EIF4E2, EIF5, FAU, PAIP1, RPL10, RPL10A, RPL11, RPL12, RPL13, RPL13A, RPL14, RPL15, RPL17, RPL18, RPL18A, RPL19, RPL23, RPL24, RPL27, RPL28, RPL29, RPL3, RPL30, RPL34, RPL35, RPL35A, RPL36, RPL37, RPL37A, RPL38, RPL39, RPL4, RPL41, RPL5, RPL6, RPL7, RPL7A, RPL8, RPL9, RPLP0, RPLP1, RPLP2, RPS11, RPS12, RPS13, RPS14, RPS15, RPS16, RPS18, RPS19, RPS2, RPS21, RPS23, RPS24, RPS25, RPS27, RPS27A, RPS28, RPS29, RPS3, RPS3A, RPS4X, RPS4Y1, RPS5, RPS6, RPS7, RPS8, RPSA
	mRNA splicing, via spliceosome (FDR *p* = 5.8E-14)	ALYREF, AQR, BCAS2, CCAR1, CDC40, CPSF3, CWC22, CWC27, DDX23, DDX39B, DDX41, DHX15, DHX9, DNAJC8, EIF4A3, FRG1, GTF2F2, HNRNPF, HNRNPM, HSPA8, HTATSF1, MAGOH, METTL14, NCBP1, NONO, NUDT21, PABPN1, PLRG1, PNN, POLR2B, POLR2K, PRPF40A, RBMX2, RNPC3, SART3, SNRNP27, SNRPA, SNRPB2, SNRPD2, SNRPD3, SNRPG, SNW1, SRSF1, SRSF11, SRSF2, SRSF3, SRSF6, UBL5, UPF3B, USP39, ZCCHC8
	T Cell receptor signaling pathway (FDR *p* = 1.5E-12)	CD28, CD3D, CD4, DENND1B, HLA-DPA1, HLA-DPB1, HLA-DRA, HLA-DRB1, IKBKB, MAP3K7, PSMA1, PSMA2, PSMA3, PSMA4, PSMA5, PSMA7, PSMB1, PSMB10, PSMB2, PSMB6, PSMB7, PSMC2, PSMC4, PSMD1, PSMD10, PSMD11, PSMD12, PSMD2, PSMD5, PSMD7, PSMD8, PSME2, PTPN22, RIPK2, RPS27A, SKP1, STOML2, TRAF6, TRAT1
Purple	mRNA splicing, via spliceosome (FDR *p* = 2.7E-02)	CELF3, CPSF1, CPSF7, CTNNBL1, DHX38, DHX8, HNRNPA2B1, HNRNPU, POLR2D, POLR2H, SNRPC, SRSF5, TRA2A, U2AF1, U2AF1L4, ZRSR2

**Fig. 7:**
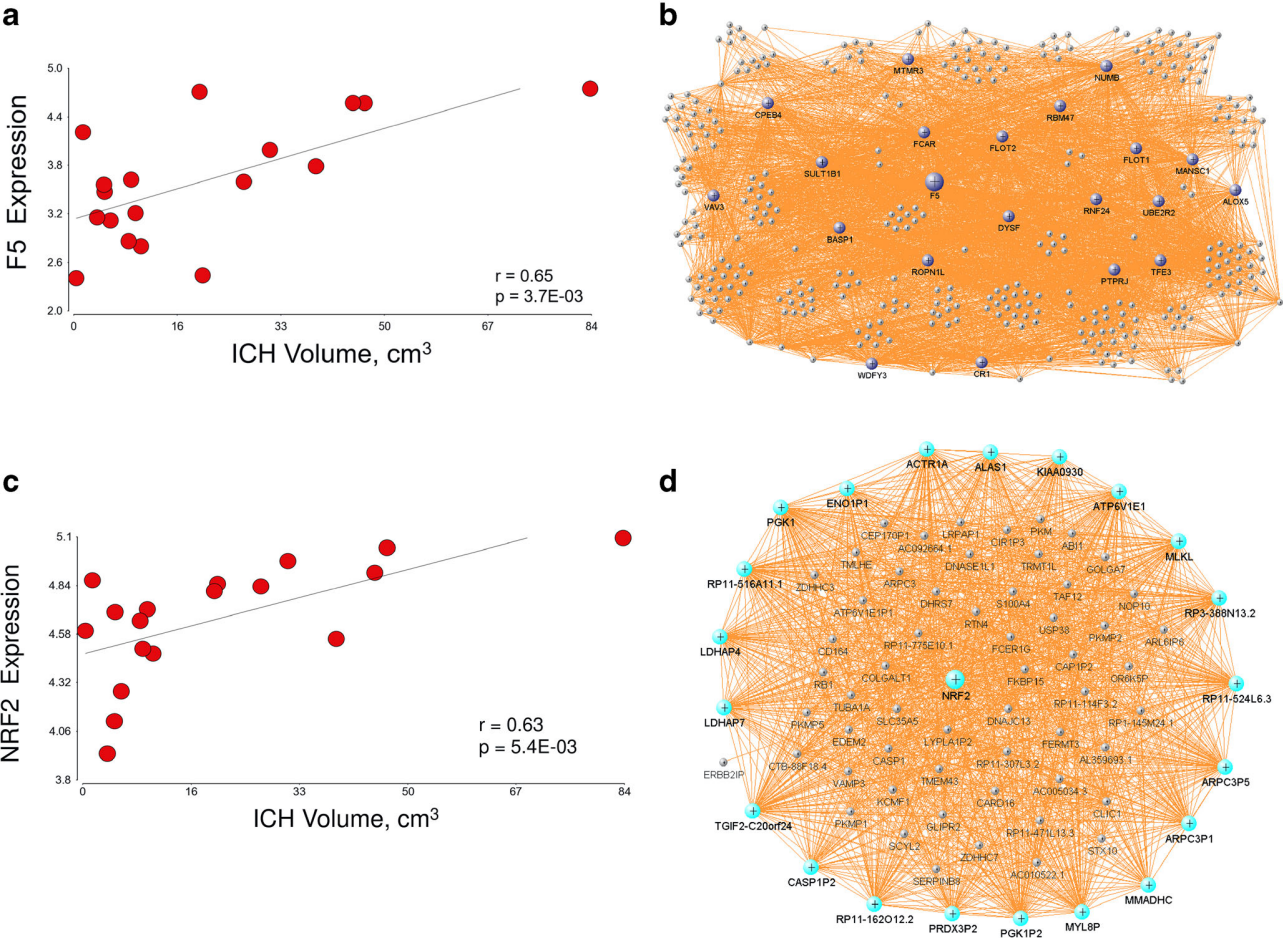
Subjects’ F5 expression correlated to their measured ICH volume (**a**). Linear correlation p and linear correlation r are presented on the figure. Partial correlation p and partial correlation r from model 1 were 6.3E-03 and 0.65, respectively. F5 network in MidnightBlue module (**b**). Genes colored in midnight blue are hub genes. Subjects’ NRF2 expression correlated to their measured ICH volume (**c**). Linear correlation p and linear correlation r are presented on the figure. Partial correlation p and partial correlation r from model 1 were 3.3E-03 and 0.69, respectively. NRF2 network in the Cyan module (**d**). Genes colored in cyan are hub genes

**Fig. 8:**
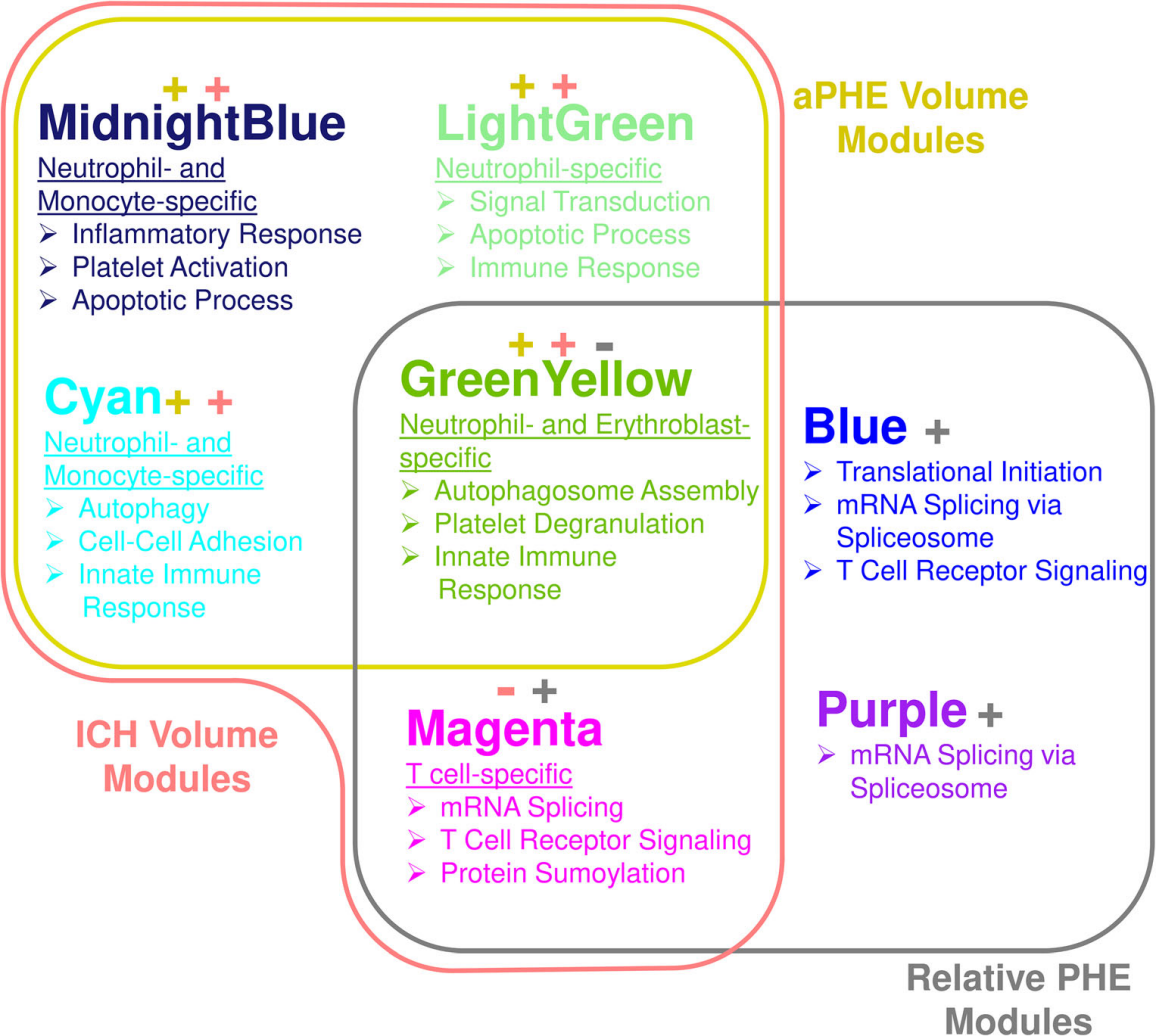
Schematic representation of the modules associated with the volumetric parameters and their top GO biological processes. The + and − signs denote the correlation direction between the eigengene of the module and the volumetric parameter (color-coded: yellow—absolute PHE volume, red—ICH volume, gray—relative PHE)

The hubs’ top GO biological processes are presented in Table 4 (FDR *p* < 0.05). MidnightBlue’s hubs were enriched in regulation of cell adhesion (FDR *p* = 1.2e-02) including the hub F5 (coagulation factor V; Fig. 7). Magenta hubs were enriched in T cell-specific genes, including LCK and ITK, which are Src family tyrosine kinases. A summary of module GO findings can be found in Fig. 8.

**Table 4:** Top GO biological process terms and their genes among the hubs of the significant modules

Module hub	GO term	Genes
MidnightBlue hubs	Positive regulation of cell adhesion (FDR p = 1.2E-02)	PTPRJ, VAV3, TFE3
GreenYellow hubs	Positive regulation of apoptotic process (FDR *p* = 3.2E-02)	BCL2L1, FGD4, MAP3K5, RASSF2, SOS2
Magenta hubs	T cell receptor signaling pathway (FDR *p* = 4.8E-02)	ITK, LCK, RFTN1, SKAP1
Blue hubs	Translation initiation (FDR *p* = 1.4E-34)	EIF3D, EIF3M, RPL13A, RPL14, RPL15, RPL19, RPL3, RPL36, RPL37, RPL4, RPL5, RPL6, RPL7A, RPS13, RPS16, RPS27, RPS27A, RPS3, RPS5, RPS6, RPS8
	mRNA nonsense-mediated decay (FDR *p* = 5.2E-31)	RPL13A, RPL14, RPL15, RPL19, RPL3, RPL36, RPL37, RPL4, RPL5, RPL6, RPL7A, RPS13, RPS16, RPS27, RPS27A, RPS3, RPS5, RPS6, RPS8
	rRNA processing (FDR *p* = 3.5E-26)	RPL13A, RPL14, RPL15, RPL19, RPL3, RPL36, RPL37, RPL4, RPL5, RPL6, RPL7A, RPS13, RPS16, RPS27, RPS27A, RPS3, RPS5, RPS6, RPS8

The hubs from the rest of the 7 significant modules do not have biological processes passing FDR *p* < 0.05)

## Discussion

We identified genes and gene modules whose expression in peripheral blood correlated with ICH volume, aPHE volume, and rPHE—all key determinants of ICH outcome [4, 16, 35]. Expression of 440, 266, and 391 genes correlated with ICH volume, aPHE volume, and rPHE, respectively. Seven modules significantly correlated with at least one volumetric measure. Five modules were enriched in neutrophil, monocyte, erythroblast, and/or T cell-specific genes. These cell types coordinate damage and repair following ICH [12]. Inflammation, autophagy, apoptosis, mRNA Splicing, and T Cell Receptor Signaling were overrepresented in the gene lists. This human data complements experimental ICH studies [12] and may help guide the search for future treatments. Additional discussion can be found in Online Resource 1.

### Genes/Modules Associated with ICH and aPHE Volumes

#### Inflammatory Genes/Modules Activated with Larger ICH and aPHE Volumes

Inflammation plays a critical role in brain damage and repair following ICH [12]. Intraparenchymal blood triggers an inflammatory cascade activating microglia to release cytokines, chemokines, reactive oxygen species, and extracellular proteases. It causes BBB dysfunction and facilitates systemic immune cell influx into the injured brain [12]. Several modules correlated with ICH and aPHE volumes were populated with activated immune/inflammatory pathways (Fig. 8).

The Leukocyte Extravasation Signaling pathway was associated with larger ICH and aPHE volumes. It included adhesion molecules that cause BBB dysfunction, including MMP9, MMP25, ICAM1, ITGAM, TIMP2, VAV3, and VASP. MMP9 causes BBB dysfunction resulting in increased capillary permeability and brain edema following ICH [12, 36]. Depletion of neutrophils, the main MMP9 source, reduced BBB break-down and decreased PHE [12, 37]. The ICAM1 adhesion molecule, induced within hours of experimental ICH [38, 39], promotes leukocyte adhesion which causes neuronal injury [7]. The steroid dexamethasone reduced edema volume by reducing MMP9 and ICAM1 levels [39], though a human trial of dexamethasone did not benefit ICH [5]. TIMP2 (tissue inhibitor of metalloproteinases-2) inhibits MMPs and endothelial cell proliferation. An MMP and TIMP imbalance is postulated to cause extracellular matrix degradation leading to progression and rupture of cerebral aneurysms [40]. TIMP2 polymorphisms are associated with increased ICH risk [41]. The above genes, as well as hubs for the Leukocyte Extravasation Signaling pathway (VAV3, NCF4), may be treatment targets for reducing hemorrhage and edema volumes.

Following ICH, Toll-like receptors activate NF-kB signaling to induce factors such as TNF-α, ICAM1, high mobility group box1 (HMGB1), IL-1β, nitric oxide synthase (NOS), and heme oxygenase 1 (HO-1) [12, 42]. NF-kB activation likely occurred via overrepresented TLRs, including TLR1, TLR4, TLR5, TLR8, and TLR10 which all correlated with ICH and aPHE volumes. TLRs play key roles in immune and inflammatory responses to ICH [43]. TLR4 is known to be activated by hematoma components and induce neuroinflammation following ICH [44]. TLR4 also mediated hemorrhagic transformation following delayed tPA administration in experimental ischemic stroke [45]. TLR4-deficient mice with ICH have reduced perihematomal inflammation and improved functional outcomes [46].

Another overrepresented inflammatory pathway, HMGB1 Signaling [47], included pro-inflammatory molecules like ICAM1, TNFSF10, TNFSF13B, and TNFSF14. TNFSF/TNFRSF regulate innate and adaptive immunity and cell death [48]. HMGB1 Signaling was activated with larger ICH and aPHE volumes.

Inflammasome pathways also associated with ICH and aPHE volumes. Inflammasomes are innate immune system complexes that interact with Pattern Recognition Receptors (PRRs) to trigger inflammation in response to infection and Damage-Associated Molecular Patterns (DAMPs) released from dying or damaged cells. Inflammasomes participate in a variety of inflammatory processes including those occurring in ICH [49]. In this study, NLRP3, NLRC4, NLRC12, AIM2, and IFI16 (interferon gamma inducible protein 16), which are major components of the NLRP3-, NLRC4-, NLRC12-type, and AIM2-type inflammasomes, positively correlated with ICH and aPHE volumes (Fig. S1,S2). The NLRP3 inflammasome amplifies inflammatory ICH responses, with NLRP3 inhibitor MCC950 being administered as an ICH treatment [44, 49–52]. NLRP3 knock-down after ICH reduced brain edema and improved neurological outcomes [51]. NLRC4, a key component of the NLRC4 inflammasome, regulates inflammation following cerebral ischemia [53]. Inhibition of the AIM2 inflammasome following subarachnoid hemorrhage (SAH) decreased GMDSD-induced pyroptosis, a form of programmed cell death [54]. Our previous study showed that NLRC4 was a hub gene for ICH compared to controls [29]. Here, we find NLRP3-, NLRC4-, NLRC12-, and AIM2-type inflammasomes correlated with ICH and aPHE volumes following human ICH.

Peroxisome Proliferator-Activated Receptor (PPAR) Signaling was suppressed in subjects with larger ICH and aPHE volumes. PPARγ agonists improve experimental ICH outcomes [55–57], and we previously documented PPARγ signaling molecules in ICH gene network analyses [15, 29]. Statins enhance hematoma clearance and improved neurological outcome in a PPARγ-dependent manner in a rat ICH model [58]. PPARγ regulates Nrf2 [56] which regulates antioxidant genes with antioxidant response elements (ARE) in their promoters. Here, NRF2-mediated Oxidative Stress Response was activated and correlated with ICH and aPHE volumes and NRF2 itself was a hub gene that correlated with ICH volume (Fig. 7). Microglial NRF2 enhances anti-oxidative capacity, phagocytosis, and hematoma clearance [59]. In addition, Fcγ Receptor-Mediated Phagocytosis in Macrophages and Monocytes correlated with lCH and aPHE volumes. These scavenger mechanisms are likely involved in hematoma resolution following ICH [55].

B Cell Signaling was overrepresented in several modules and correlated with ICH and aPHE volumes. B cell involvement in ICH pathophysiology remains unclear as few cells infiltrate ICH brain [12, 35]. However, we previously demonstrated differential B cell receptor signaling following ICH compared to controls [15, 29]. Here, we provide evidence for B cell association with ICH and aPHE volumes. Although B cells may not directly affect the ICH injury zone, they modulate other immune cells that could affect ICH and aPHE volumes [60].

Neuroinflammation Signaling was overrepresented in several modules and is a major contributor to ICH injury [12, 35]. Activated genes included MMP9, ICAM1, TLR1,4,5,8,10, GSK3B, NCF1, NCF2, CREB, and CREBBP (Table S2). The CFLAR and NCF2 hub genes correlated positively with ICH and aPHE volumes, and negatively with rPHE (Fig. S3). CFLAR is an apoptosis regulator, linking cell survival and cell death pathways [61]. It promotes vascular smooth muscle cell survival and is upregulated by NOTCH3, mutations in which cause CADASIL1, a genetic cause of stroke [62]. NCF2 (neutrophilic cytosolic factor 2), also known as p67 phagocytic oxidase (p67phox), is the cytosolic subunit of the NADPH oxidase (NOX) complex found in neutrophils which produces a burst of superoxide. Inhibition of NOX family members protects against ischemic and traumatic brain injury [63]. Thus, this hub gene/network is a potential ICH treatment target.

#### Autophagy Pathways Associated with ICH and aPHE Volumes

Autophagy involves lysosome-dependent degradation/recycling of cellular components. The autophagosome, a double-membrane bound organelle that fuses with lysosomes for digestion, involves recruitment of autophagy-related (ATG) and associated proteins [64]. Autophagy plays a role in homeostasis, cell death, and immunity/inflammation [64]. Autophagy dysregulation is implicated in cardiovascular diseases, neurodegenerative diseases, ischemic stroke, TBI, SAH, and ICH [65, 66]. Iron overload from the hematoma plays a key role in ICH-induced autophagy, which can be reduced by iron chelation [67]. Autophagy may also regulate ICH-induced neural damage through apoptosis and the NF-kB signaling pathway, with autophagy suppression being protective in experimental ICH [68]. This study shows autophagy was associated with ICH and aPHE volumes, and autophagosome assembly was positively associated with ICH and aPHE volumes but negatively with rPHE. This included major autophagy genes including ATG3, part of the autophagosome essential for autophagic cargo delivery to lysosomes [64]. Another autophagy-related gene, BECN1 (Beclin 1, aka ATG6, autophagy-related 6 homolog) regulates autophagy. Autophagy interacts with inflammatory signaling pathways though IKK-NFkB [64]. NF-kB induces autophagy by transactivating BECN1 and inhibits autophagy through TNFα-associated mechanisms [64]. Clathrin-mediated endocytosis signaling was also enriched, a process that involves intracellular trafficking which links endocytosis to autophagy [69]. These pathways all participate in hematoma clearance [59]. Autophagy can be beneficial or detrimental by triggering either pro-survival or pro-death mechanisms; thus, more studies are needed to elucidate its roles in human ICH.

#### Cell Death Pathways Associated with ICH and aPHE Volumes

Numerous factors trigger programmed cell death (PCD) following ICH in perihematomal and distant brain regions [4, 70, 71]. PCD involves apoptosis, autophagy, pyroptosis, necroptosis, and ferroptosis, while necrosis is considered non-programmed cell death [70, 71]. A portion of brain damage post-ICH is due to apoptosis [4, 72]. Apoptosis signaling was associated with ICH and aPHE volumes. PTEN Signaling, which promotes apoptosis [73, 74], was suppressed with larger ICH volumes. PTEN inhibition is neuroprotective following experimental ICH [75]. Blood Cell Viability correlated with ICH volume, and pyroptosis of macrophages [70] showed a trend toward activation (Table S7A). Pyroptosis is also implicated in post-ICH brain injury [71]. Pyroptosis, unlike apoptosis, is an inflammatory form of PCD initiated by nucleotide-binding oligomerization domain-like receptors, NLRs, which recognize pathogen-associated molecular patterns (PAMPs) in the cytosol [71]. Upon ligand binding, these receptors initiate assembly of an NLR-based multiprotein complex, the Inflammasome, like NLRP3 mentioned above. Necrosis showed a trend toward suppression, while Cell Survival was activated with larger ICH and aPHE volumes (Table S7). Our data suggests that many different cell death mechanisms are associated with hematoma and aPHE volumes following ICH.

#### Growth Factor Signaling Pathways Associated with ICH and aPHE Volumes

Growth factors (GFs) have been implicated in ICH pathophysiology [36]. There were 24 overrepresented growth factor signaling pathways including Vascular Endothelial Growth Factor (VEGF), Granulocyte-Macrophage Colony-Stimulating Factor (GM-CSF), Transforming Growth Factor Beta (TGF-β), Hepatic Growth Factor (HGF), Fibroblast Growth Factor (FGF), Nerve Growth Factor (NGF), Neuregulin, Neurotrophin/TRK, and others (Fig. 6). High serum levels of VEGF, angiopoetin-1 (Ang-1), and G-CSF have been associated with good functional outcome following human ICH [36]. G-CSF has been investigated as a potential ICH treatment to reduce PHE, BBB permeability, and improve recovery [36]. In this study of peripheral blood leukocytes, GM-CSF Signaling, including a CSF receptor subunit CSF2RB, was activated with larger ICH and aPHE volumes. CSF3R, another GM-CSF receptor, was a hub in LightGreen, though GM-CSF Signaling was not overrepresented in this module (Fig. S4). VEGF reduced brain edema, neuronal loss, and neurological deficits in experimental ICH [76]. However, increased VEGF exacerbated hemorrhage after experimental brain arteriovenous malformations [77]. One study showed higher VEGF serum concentrations within 72 h in subjects with larger ICH volume (> 30 cm^3^) and higher ICH severity; however, when stratified by good vs. poor outcome at 90 days, subjects with higher VEGF levels had better outcome [78]. In our study, VEGF Signaling was activated following larger ICH and aPHE volumes. The timing of pathway activation may determine whether VEGF mediates injury or repair. TGF-β Signaling was another GF pathway associated with ICH and aPHE volumes, a well-described cascade that controls cell differentiation, proliferation, migration, and apoptosis. In a murine ICH model, TGF-β1 treatment increased TGF-β1 pathway activation in microglia and improved functional outcome [79]. Furthermore, increases in plasma concentration of TGF-β1 from 6 to 72 h after human ICH has been associated with improved 90-day outcomes [79]. PDGF Signaling, implicated in neuroinflammation after experimental ICH [80], was also correlated with ICH volume. Activation of GF signaling pathways in peripheral leukocytes of subjects with larger ICH and aPHE volumes may decrease injury and enhance repair. However, given their diverse effects, they may also contribute to brain injury particularly early [81].

#### Coagulation, Platelet, and Cardiovascular Pathways Associated with ICH and aPHE Volumes

Coagulation and platelet activation are involved in ICH pathophysiology and have been investigated as treatment targets for hematoma expansion [82]. Coagulation and platelet disorders are a risk factor for ICH and worsen outcome [83]. The coagulation cascade, involving intrinsic and extrinsic pathways, results in thrombin activation and fibrin formation from fibrinogen [82]. In this study Thrombin and Thrombopoietin (TPO) Signaling pathways were activated with larger ICH and aPHE volumes. TPO (gene symbol THPO) is a humoral growth factor that stimulates platelet formation, reduces ischemic brain injury, and improves outcome partly by inhibiting MMP9 and BBB dysfunction [84]. TPO-receptor agonists are used to treat ICH associated with severe immune thrombocytopenia, a condition with accelerated platelet destruction and impaired platelet production [85]. Platelet aggregation was activated through the Thrombin Signaling Pathway in larger ICH and aPHE volumes. Finally, the factor 5 (F5) hub gene, associated with hematoma and aPHE volumes (Fig. 7), is a non-proteolytic co-factor of factor X and an essential part of the prothrombinase complex [86], and may modulate ICH and aPHE volumes.

Platelets are major effector cells in hemostasis and thrombosis [86], promote leukocyte recruitment to the vascular injury site, and contribute to vasoconstriction post ICH [87]. Platelets interact with collagen from blood and activate leukocytes leading to platelet degranulation, which involves serotonin (5-HT) exocytosis and increased vascular permeability [87]. Reduced platelet activity may be associated with early hematoma growth and worse outcomes [82]. All of these, plus the fact that one-third of ICH cases occur in patients on antiplatelet drugs, suggest that platelet function in ICH and hemorrhage expansion is an important factor [82]. In this study, platelet activation and platelet degranulation correlated with ICH and aPHE volumes (Fig. 8). This included G protein coupled receptors (S1PRs) which interact with sphingosine-1-phosphate (S1P), involved in maintaining BBB integrity [82]. S1PR1 (sphingosine-1-phosphate receptor 1) was in Magenta, a module negatively correlated with ICH volume and positively with rPHE. S1PR4 was in Cyan, a module positively correlated with ICH and aPHE volumes. GP1BB (glycoprotein Ib platelet subunit beta), a part of the GPIb-V-IX system of the receptor for von Willebrand factor (VWF) which mediates platelet adhesion [82] was also in Cyan. PPBP (pro-platelet basic protein, aka CXCL7 and low affinity platelet factor IV (PF4)), in a module positively correlated with ICH and aPHE volumes and negatively with rPHE, is a platelet-derived growth factor and a platelet-specific chemokine implicated in thrombosis, thrombocytosis, and heparin-induced thrombocytopenia [88].

Several cardiovascular function pathways were activated with larger ICH and aPHE volumes, including Renin-Angiotensin Signaling and Apelin Endothelial Signaling. Renin-angiotensin is involved in vasoconstriction/vasodilation, which was activated in this study. Apelin endothelial signaling is involved in angiogenesis, endothelial cell mitosis, and promoting endothelial cell assembly and proliferation. The pathway is induced by hypoxia and sheer stress. The acute effect of apelin signaling in the mature, intact endothelium is vasodilation and promotion of vessel formation. In our study, angiogenesis, cell proliferation, and inflammation were predicted activated through this pathway. Our previous studies also implicated angiogenesis following ICH [15, 29], likely an important component of ICH repair.

#### T Cell Receptor Signaling and Other T Cell Pathways Negatively Correlated with ICH Volume and Positively with rPHE

One module (Magenta) and its hubs that negatively correlated with ICH volume but positively with rPHE was enriched in T cell receptor signaling and T cell-specific genes (Fig. 4, Fig. S5). Several overrepresented T cell pathways were suppressed with larger ICH volumes, including PKCθ Signaling in T Lymphocytes, CD28 Signaling in T Helper Cells, iCOS-iCOSL Signaling in T Helper Cells, Calcium-Induced T Lymphocyte Apoptosis, and Th1 Pathway. T cell-specific hub genes included TRAJ36 (a T cell receptor), ITK, LCK, S1PR1, and SKAP1 (Fig. S6-S10). LCK is expressed in T cells [24], involved in selection and maturation of developing T cells [89, 90], and a member of the Src family kinase (SFK) gene family. We have shown that SFK inhibition improved outcomes and BBB function following experimental ICH [89, 90]. SKAP1 (Src kinase associated phosphoprotein 1) interacts with SFKs and stimulates T cell antigen receptors to activate integrins. ITK (IL2-inducible T cell kinase) is involved in T cell proliferation and differentiation. S1PR1, though not a hub itself, only had hub genes in its network (Fig. S9). S1PR1 is a target of fingolimod, an FDA-approved immunosuppressive drug for multiple sclerosis. Fingolimod targets its receptors S1PR1, 3, 4, and 5, downregulates S1PR1 on T cells, and inhibits S1PR1-dependent lymphocyte egress [91]. A pilot ICH study showed it reduced perihematomal edema and neurologic impairment [35, 91]. Other S1PR1 agonists are being investigated as ICH treatments. Currently, an ICH phase 2 clinical trial of Siponimod, a dual agonist at S1PR1 and S1PR5, is ongoing (ClinicalTrials.gov Identifier: NCT03338998). T Cell Receptor Signaling genes from the per-gene analyses were different from Magenta’s genes and were mainly positively correlated with ICH and aPHE volumes, including MAPK1, MAPK3, NFKBIA, PIK3CG, RALB, and VAV3.

Eight hundred thirty-seven genes out of the 1225 genes that we previously showed differentially expressed in ICH versus controls [29] were found to be associated with the volumetric measures in this study. Of the 71 T cell receptor genes identified in this study, 49 were identified in our previous studies [15, 29]. In those studies, T cell receptor transcripts and genes were downregulated in ICH peripheral blood compared to controls [15, 29] and differentiated ICH from ischemic stroke [15]. Thus, T cell receptor genes and other T cell genes are differentially expressed and differentially spliced in ICH compared to controls and ischemic stroke [15, 29], but also correlate with ICH and perihematomal edema volumes and may be viable biomarkers and therapeutic targets.

### Genes/Modules Associated with rPHE

Since ICH and aPHE volumes are highly correlated [16] (*r* = 0.97, *p* = 7.7e-11 in our dataset), differentiating molecular correlates between ICH and aPHE is challenging (Fig. S11). We calculated the rPHE to highlight commonalities as well as distinct processes that determine ICH and PHE volumes. rPHE can be large when ICH volume is small and may not necessarily correlate with clinical outcome [16]. There was a weak non-significant negative correlation between aPHE and rPHE (*r* = − 0.27, *p* = 2.8e-01), driven in part by subjects with small aPHE volume relative to the ICH volume, and by subjects with large aPHE volume relative to the ICH volume (Fig. S12). There was a small overlap between genes (Fig. 1a) and modules (Figs. 4a and 8) associated with aPHE and rPHE.

#### Genes Correlating with rPHE

Three hundred ninety-one genes correlated with rPHE, with many being neutrophil-specific genes. Specific biofunctions included chemotaxis of neutrophils, which included genes with strong negative correlations between rPHE and AQP9 (aquaporin 9), CXCR1, CXCR2, and TREM1, and strong positive correlations with CXCL2 and CCL3L1 (Table S7). Aquaporins are water channel proteins that participate in water homeostasis and edema formation, and have been ICH treatment targets [92]. AQP9 was also a hub gene, had a sub-network (Fig. S13) including TLR1, TLR4, CFLAR, and NCF2, and was significantly enriched in neutrophil-specific genes (Table S4R). Another aquaporin, AQP3, was in a module positively correlated with rPHE. TREM1 stimulates pro-inflammatory molecules and amplifies neutrophil- and monocyte-mediated inflammation. CXCL2 exacerbates inflammatory responses to ICH [12]. rPHE-correlating genes were also overrepresented in JNK Cascade and Mitochondrial Translation Elongation. JNK pathway inhibition reduces inflammation and edema following ICH [93–95]. Several genes associated with rPHE (AQP9, CXCL2, DLG4, FPR1, NR1D1, PQLC1, PRSS3, SIN3B, TRAK1, VAMP8) were differentially expressed in perihematomal human brain following ICH [96], further supporting their importance for edema pathophysiology.

#### Protein Translation, Stress Response Pathways, and Necroptosis Associated with rPHE

The Blue module, positively correlated with rPHE, was overrepresented by major protein translation, stress response, and cell death pathways, including Eukaryotic Initiation Factor (EIF), p70S6K, mTOR, Nucleotide Excision Repair (NER), protein ubiquitination, Unfolded Protein Response (UPR), Endoplasmic Reticulum Stress Pathway, and Necroptosis Signaling (Fig. 5f, Table S2O)—many of which have been implicated in ICH [97]. The inability to properly fold proteins leads to UPR and often programmed cell death including necroptosis [65, 97]. TNF-α and receptor-interacting serine/threonine-protein kinase 1 (RIPK1) trigger necroptosis and inflammation. RIPK2, a member of the Blue module associated with rPHE, is a key regulator (in combination with RIPK1 and RIPK3) of inflammatory signaling and cell-death pathways [98, 99]. Inhibition of RIPK1 reduced neuronal death and improved functional outcome following experimental ICH [100]. TNFAIP8 (TNF alpha-induced protein 8), a negative mediator of apoptosis [101], and HMGB1 also correlated with rPHE. HMGB1 is released by microglia following cell injury and death [102], and participates in ICH-induced inflammatory injury [44]. An HMGB-1 inhibitor attenuates ICH-induced injury in experimental ICH [102, 103].

Molecules in the NER Signaling pathway included 4 (COPS2,4,6,8) of the 8 subunits of the COP9 signalosome (CSN) complex [104], and NEDD8, a ubiquitin-like protein modifier. CSN controls protein degradation, serves as docking platform for signaling molecules, and has been associated with atherosclerosis, ischemic stroke, and protecting blood vessels and the heart [104]. rPHE was also associated with Translation Initiation, mRNA Non-Sense Mediated Decay and rRNA Processing, including many ribosomal proteins. Thus, RNA and protein metabolism contribute to rPHE following ICH.

#### mRNA Splicing Pathways Associated with rPHE

mRNA splicing is the process by which one gene produces multiple transcripts and thus multiple protein isoforms, and correlated with rPHE (Fig. 8). RNA mis-splicing is implicated in many diseases [105]. rPHE correlated with components of the spliceosome, the ribonucleoprotein complex that performs pre-mRNA splicing. For example, SNRPA (Blue module member) is a component of the spliceosomal U1 small nuclear ribonucleoprotein (snRNP) subunit, one of 5 U-snRNP subunits which together with 80 additional proteins constitute the spliceosome. SNRPA is essential for pre-mRNA 5′ splice-site recognition and subsequent spliceosome assembly [105]. Additional spliceosome core components from the rPHE modules included SNRPB, SNRPB2, SNRPD2, SNRPD3, SNRPF, SNRPG, SNRPC, and SNRPN. rPHE also correlated with the Spliceosomal Cycle (activated with larger rPHE) and Mediator Complex components (7/30 including MED1, MED11, MED17, MED21, MED27, MED29, MED30) [106, 107]. The Mediator Complex interfaces between gene-specific transcription factors and RNA polymerase II, which transcribes all mRNA, most small nuclear RNA, and miRNA [107]. It bridges gene-specific regulatory signals to convey information to the basal RNA-Pol II machinery, thus controlling cell physiology, growth, development and differentiation [107]. It has been implicated in many diseases including cardiovascular disorders [107]. Additionally, SMARCE1, part of the chromatin remodeling complex SWI/SNF and required for transcriptional activation via epigenetic regulation, was a Blue hub. We have previously shown differentially spliced genes and expression modules in human ICH which differentiated ICH from controls and ischemic stroke [15, 29, 108]. These previous studies, along with the current findings, underscore the significance of RNA and protein metabolism, splicing, and epigenetic mechanisms [109, 110] in ICH pathophysiology.

### Genes Implicated in ICH Genetics and Overlap with Previous Brain Studies

We overlapped our lists of genes associated with hematoma and edema volumes with genes implicated in ICH genetics [8] and found many overlaps: solute carrier family 25 member 44 (SLC25A44), amyloid beta precursor protein (APP), gelsolin (GSN), polyamine modulated factor 1 (PMF1), and prion protein (PRNP) (Fig. 4a, Table S4S). Thus, these genes may have a causal relationship determining ICH and aPHE volumes, as well as rPHE. We next compared our hematoma and edema volume gene lists to genes differentially expressed in human perihematomal brain tissue [96]. There was a significant overlap with ICH volume (27 genes, *p*(overlap) = 4.0e-05), aPHE volume (12 genes, *p*(overlap) = 4.7e-02), and with three modules (Fig. 4a, Table S4T). Although expressed in blood and brain, these genes might have different functions in the two organs.

### Limitations

Due to small sample size, the results from this study need to be validated in a larger cohort. The cell-specific genes used here [23, 24] were identified in healthy subjects and may change expression in other cells with disease [111–114]. Since hematoma and PHE volumes are major determinants of ICH outcomes, the genes and networks identified here should be studied with respect to outcomes in future studies. It is possible that some of the gene expression differences correlated with the volumetric measures in this study are actually a result of changes of cell counts of particular cell types. Future studies on isolated cell subtypes will need to determine the contribution of changes of cell proportions to the observed gene expression findings. In addition, sex and age are important factors in ICH pathology and pathophysiology [1, 115–117]. Despite there being no age or sex significant modules, some individual genes within these modules may have been impacted by these factors. Future studies in larger sample sizes will have to investigate gene expression affected by sex and age interactions associating with the ICH volume, aPHE volume, rPHE size, and outcome.

## Electronic Supplementary Material

ESM 1 Supplemental Methods and Discussion (PDF 177 kb)

ESM 2 Supplemental Figures (PDF 2218 kb)

ESM 3 Supplemental Tables (XLSX 1.32 mb)

ESM 4 Captions for Supplemental Material (PDF 82.4 kb)

## Data Availability

The data generated for this study will be available upon reasonable request.
