# Supplementary material for: Molecular Correlates of Hemorrhage and Edema Volumes Following Human Intracerebral Hemorrhage Implicate Inflammation, Autophagy, mRNA Splicing, and T Cell Receptor Signaling
Source: Transl Stroke Res. 2020 Nov 18;12(5):754–77. doi: 10.1007/s12975-020-00869-y (PMC8421315; doi:10.1007/s12975-020-00869-y)
Supplement: Supplementary file 1 — Supplemental Methods and Discussion (PDF 177 kb) [file 12975_2020_869_MOESM1_ESM.pdf]

Supplemental Material

for

Molecular Correlates of Hemorrhage and Edema Volumes following Human Intracerebral Hemorrhage Implicate Inflammation, Autophagy, mRNA Splicing, and T Cell Receptor Signaling

Translational Stroke Research

Marc Durocher[1]*, Bodie Knepp[1]*, Alan Yee[1], Glen Jickling[1,2], Fernando Rodriguez[1], Kwan Ng[1], Xinhua Zhan[1], Farah Hamade[1], Eva Ferino[1], Hajar Amini[1], Paulina Carmona-Mora[1], Heather Hull[1], Bradley P. Ander[1], Frank R. Sharp[1] and Boryana Stamova[1]

*co-first authors

[1]Department of Neurology, School of Medicine, University of California at Davis, Sacramento, CA, USA.

[2]Department of Medicine, University of Alberta, Edmonton, Canada.

Corresponding Author: Boryana Stamova (bsstamova@ucdavis.edu)

Supplemental Methods

Study Subjects

The study protocol was approved by UC Davis Institutional Review Board and adheres to all federal and state regulations related to the protection of human research subjects, including The Common Rule, the principles of The Belmont Report, and Institutional policies and procedures. Written informed consent was obtained from all participants or their proxy at the University of California Davis (UCD) Medical Center. Intracerebral Hemorrhage (ICH) diagnoses were conducted by board-certified neurologists based upon medical histories, exams, computed tomographic (CT) brain scan and/or magnetic resonance imaging (MRI). ICH locations and patient demographics are listed in Table 1. Exclusion criteria included hemorrhagic transformation following ischemic stroke, subarachnoid hemorrhage (SAH), traumatic brain injury (TBI), recent infection (<2 weeks), chronic infection (like HIV), immunosuppressive therapy, and cancer

Blood Collection, RNA Isolation

Peripheral venous blood was collected in PAXgene tubes from ICH subjects and RNA isolated and prepared for hybridization on GeneChip® Human Transcriptome Array (HTA) 2.0 as previously described[1]. There was a single blood draw per subject, which varied from 4.2 to 124.3 hours from symptom onset (Table 1). Due to the wide range of times and the temporally dynamic peripheral immune response following ICH, time was considered in the analyses.

Arrays and Processing

Total RNA was prepared for hybridization on the GeneChip® Human Transcriptome Array (HTA) 2.0 (Affymetrix, Santa Clara, CA). This array allows for examination of the coding and non-coding human transcriptome. Please note the HTA 2.0 array targets precursor (immature) microRNAs rather than the mature species. GC Correction (GCCN) and Single Space Transformation (SST) were applied to the HTA .CEL files using Affymetrix Power Tools (APT). GCCN-SST transformed *.CEL* files were then uploaded into Partek Flow software (Partek Inc, St. Louis, MO) where probe sets were mapped to the Human Genome hg19 using the STAR 2.4.1d aligner. Nominal read coverage depth was defined as 30 million and default mapping parameters were used. Quantification to transcriptome (Ensembl75), RPKM normalization and an offset of 1.0 accounting for the zero values was applied. This summarized gene-level expression was then imported into WGCNA for co-expression network analysis or analyzed in Partek Genomics Suite (Partek Inc, St. Louis, MO) in per-gene regression models.

56,358 genes present in the data were filtered by removing genes with a maximum expression across all samples of <5, and with reads <3 in > 50% of the male and female samples across all groups, resulting in a final dataset of 21,175 genes. This was done to remove potential outliers, reduce noise, and prevent sex bias due to an uneven number of males and females[2].

ICH and PHE Volume Measurements

ICH and PHE volume measurements were conducted on subjects' CT Brain scans using AnalyzePro 1.0 software (AnalyzeDirect, Inc.). CT scans were taken after symptom onset and prior to blood draw. Slice thickness was 2.5 mm. Image intensity was set to the default specifications for T1 weighted CT Brain images. To isolate objects (skull, ICH, edema), we used the "semi-automatic" option with the "region grow" option sub-selected. This option allowed for the isolation of objects using a "seed point" and manually selected edge strength minimum and maximum gradient thresholds. A 2-D object boundary was then created and the specifications automatically applied to each slice. At times it was necessary to manually adjust the object boundary, using the "manual" option. Once the three objects (skull, ICH, and PHE) were isolated and locked, the images were opened in the "measure" module, where the 2-D images were modeled into 3-D objects. Here, each object could be selected separately, and the volume of the selected object displayed in cm^3. The absolute PHE volume, which refers to the sum total of pixels of the PHE lesion[3] is referred to as aPHE volume for simplicity. Subjects with large ICH volumes may also have large aPHE volumes due to the ICH's large circumference, even if the PHE itself is not very large. To adjust for this, we calculated the ratio between the aPHE and ICH volumes to estimate the relative PHE size (rPHE, a unitless ratio) as described in Selim and Norton, 2020[3].

Network Visualization

The R function within WGCNA *visantPrepOverall* was utilized to generate a list of intramodular gene connections that could be imported into VisANT to visualize the

networks[4, 5]. To most effectively capture hub gene connectivity, parameters modified within the *visantPrepOverall* function were numint=10,000 and signed=TRUE. The minimum weight cutoff for each network was adjusted within VisANT to display a visually distinguishable number of connections. For the images provided in this document, the lengths and colors of the edges (connections) are arbitrary as are the location of the nodes (genes). Networks were created in VisANT for modules deemed significant with p(ICHvol)<0.05, p(aPHEvol)<0.05, or p(rPHE)<0.05 and for subnetworks within these modules. Subnetworks contain only nodes directly connected to a given gene of interest within its assigned module.

Supplemental Discussion

Below is additional Discussion that we could not fit in the main manuscript due to space constraints.

Enrichment with Neutrophil-, Monocyte-, and T Cell-Specific Genes

Many genes that correlated with ICH and aPHE volumes were neutrophil-specific. Many hub genes were neutrophil-specific including NCF2, NCF4, STX3 and CSF3R, which activate immune responses, autophagy, neutrophil chemotaxis, and suppress lymphocyte cytotoxicity (Figure S14). Significant pathways included NF-kB Signaling (Figure S15), TREM1 Signaling and Neuroinflammation Signaling.

Neutrophils infiltrate perihematomal brain and hematoma within hours post *ictus*[6, 7]. In the early phases they contribute to BBB breakdown, microglial activation, and release of cytotoxic molecules that contribute to PHE[6, 8, 9]. Drugs that target

neutrophil-specific genes have been investigated as ICH treatment targets, including G-CSF (Granulocyte-Colony Stimulating Factor, aka CSF3)[8, 9]. CSF3R (Colony Stimulating Factor 3 Receptor) was a hub gene in this study that controls granulocyte production, maturation, and function. It is associated with Autosomal Recessive Severe Congenital Neutropenia due to *Csf3r* Deficiency and with neutrophilic leukemia, which can result in severe hemorrhage[10]. Neutrophils are a major source of MMPs, of which MMP9 and MMP25 correlated with ICH/aPHE volumes in this study. MMP inhibitors including minocycline have been shown to decrease inflammation and improve outcomes in experimental ICH[6, 8].

Genes in several modules correlating with ICH and aPHE volumes were monocyte-specific[11]. Peripheral monocytes infiltrate ICH brain within 12 hours, eventually outnumber neutrophils[8], and together with activated microglia become the major sources of pro-inflammatory IL-1, IL-6 and TNFα[6]. Monocyte-specific genes associated with ICH and aPHE volumes included cathepsin B, cathepsin Z, Heme Oxygenase 1, versican, leukocyte immunoglobulin like receptor B1, LRP1, LDL receptor related protein 1, CYP1B1, ficolin 1, P2RX7, and CTSB. Gene polymorphisms in versican have been associated with intracranial aneurysms[12] (Figure S16). Heme oxygenase 1 catalyzes heme degradation and is rapidly up-regulated in microglia following experimental ICH[13] (Figure S17). Heme oxygenase inhibitors reduce inflammation and ICH-induced PHE[8]. In our previous human study, heme oxygenase 2 showed decreased expression over time in blood following ICH[1].

As noted above, many T cell receptor and other T cell genes correlated with ICH volume and rPHE[14]. Increasing evidence suggests T lymphocytes' important role in

ischemic brain damage, however little is known about their role in ICH[8, 15]. CD4+ T cells predominate following ICH with pro-inflammatory γδT and immunosuppressive Treg also detectable[15]. In the current study, there were many T cell hub genes including RASGRP1, RAD50, LCK, ITK, KCNA3, FBXO21, and SKAP1 (Figure S7,S8,S10,S18-S21). Some of these have been ICH treatment targets. For example, fingolimod upregulates peripheral Treg cells, which inhibits microglia activation and reduces inflammatory injury and improves ICH outcome[15]. The current data suggest an important role for T cells in hematoma volume and rPHE.

Non-coding RNA Associated with ICH and aPHE volumes and rPHE

Non-coding RNA (ncRNA) have been implicated in many diseases, including ICH[16]. We have previously shown differential expression of miRNA and their putative mRNA targets in whole blood following human ICH[17]. The current study confirms that many miRNA precursors, snoRNA, and lincRNA are associated with ICH and aPHE volumes, as well as rPHE. Most of the ncRNA were miRNA. The ncRNA hubs included mir-203, mir-451b, mir-3160-1, mir-4699, LINC01167 (lincRNA), and mir-4426.

Though only precursor miRNA can be investigated on the HTA array, it is notable that mature forms of several precursor miRNAs from this study have been implicated in ICH. miRNA-223 downregulated the NLRP3 inflammasome, reduced brain edema and improved neurological function following experimental ICH[18, 19]. It is proposed as a potential serum diagnostic biomarker together with miR-181b following human ICH[20]. miR-181b also regulates ER stress-induced neuronal death in experimental ICH[21]. The data support a role for ncRNA in hematoma and edema volumes.

Association of Volumetric Measures with Clinical Parameters

Some modules that correlated with hematoma and edema volumes correlated with the neutrophil-to-lymphocyte ratio (NLR), which has been associated with worse ICH outcome[22]. Similarly, other modules correlated with lymphocyte-to-monocyte ratios (LMR) which have also been associated with ICH outcome[23]. In addition, there were other modules that correlated with hematoma and aPHE volumes that also correlated with higher WBC count, higher absolute neutrophil count and lower absolute lymphocyte count, all of which have been associated with ICH outcomes[23].

References

1. Stamova B, Ander BP, Jickling G, Hamade F, Durocher M, Zhan X et al. The intracerebral hemorrhage blood transcriptome in humans differs from the ischemic stroke and vascular risk factor control blood transcriptomes. J Cereb Blood Flow Metab. 2019;39(9):1818-35. doi:10.1177/0271678X18769513.
2. Langfelder P, Horvath S. WGCNA package FAQ. 2017. https://horvath.genetics.ucla.edu/html/CoexpressionNetwork/Rpackages/WGCNA/faq.html.
3. Selim M, Norton C. Perihematomal edema: Implications for intracerebral hemorrhage research and therapeutic advances. J Neurosci Res. 2020;98(1):212-8. doi:10.1002/jnr.24372.
4. Hu Z, Snitkin ES, DeLisi C. VisANT: an integrative framework for networks in systems biology. Brief Bioinform. 2008;9(4):317-25. doi:10.1093/bib/bbn020.
5. Miller JA, Horvath S, Geschwind DH. Divergence of human and mouse brain transcriptome highlights Alzheimer disease pathways. Proc Natl Acad Sci U S A. 2010;107(28):12698-703. doi:10.1073/pnas.0914257107.
6. Lim-Hing K, Rincon F. Secondary Hematoma Expansion and Perihemorrhagic Edema after Intracerebral Hemorrhage: From Bench Work to Practical Aspects. Front Neurol. 2017;8:74. doi:10.3389/fneur.2017.00074.
7. Zhao X, Sun G, Zhang H, Ting SM, Song S, Gonzales N et al. Polymorphonuclear neutrophil in brain parenchyma after experimental intracerebral hemorrhage. Transl Stroke Res. 2014;5(5):554-61. doi:10.1007/s12975-014-0341-2.
8. Mracsko E, Veltkamp R. Neuroinflammation after intracerebral hemorrhage. Front Cell Neurosci. 2014;8:388. doi:10.3389/fncel.2014.00388.
9. Tschoe C, Bushnell CD, Duncan PW, Alexander-Miller MA, Wolfe SQ. Neuroinflammation after Intracerebral Hemorrhage and Potential Therapeutic Targets. J Stroke. 2020;22(1):29-46. doi:10.5853/jos.2019.02236.
10. Mitsumori T, Komatsu N, Kirito K. A CSF3R T618I Mutation in a Patient with Chronic Neutrophilic Leukemia and Severe Bleeding Complications. Intern Med. 2016;55(4):405-7. doi:10.2169/internalmedicine.55.5059.
11. Watkins NA, Gusnanto A, de Bono B, De S, Miranda-Saavedra D, Hardie DL et al. A HaemAtlas: characterizing gene expression in differentiated human blood cells. Blood. 2009;113(19):e1-9. doi:10.1182/blood-2008-06-162958.
12. Sathyan S, Koshy LV, Balan S, Easwer HV, Premkumar S, Nair S et al. Association of Versican (VCAN) gene polymorphisms rs251124 and rs2287926 (G428D), with intracranial aneurysm. Meta Gene. 2014;2:651-60. doi:10.1016/j.mgene.2014.07.001.
13. Li QQ, Li LJ, Wang XY, Sun YY, Wu J. Research Progress in Understanding the Relationship Between Heme Oxygenase-1 and Intracerebral Hemorrhage. Front Neurol. 2018;9:682. doi:10.3389/fneur.2018.00682.
14. Chtanova T, Newton R, Liu SM, Weininger L, Young TR, Silva DG et al. Identification of T cell-restricted genes, and signatures for different T cell responses, using a comprehensive collection of microarray datasets. J Immunol. 2005;175(12):7837-47. doi:10.4049/jimmunol.175.12.7837.
15. Klebe D, McBride D, Flores JJ, Zhang JH, Tang J. Modulating the Immune Response Towards a Neuroregenerative Peri-injury Milieu After Cerebral Hemorrhage. J Neuroimmune Pharmacol. 2015;10(4):576-86. doi:10.1007/s11481-015-9613-1.
16. Li L, Wang P, Zhao H, Luo Y. Noncoding RNAs and Intracerebral Hemorrhage. CNS Neurol Disord Drug Targets. 2019;18(3):205-11. doi:10.2174/1871527318666190204102604.
17. Cheng X, Ander BP, Jickling GC, Zhan X, Hull H, Sharp FR et al. MicroRNA and their target mRNAs change expression in whole blood of patients after intracerebral hemorrhage. J Cereb Blood Flow Metab. 2020;40(4):775-86. doi:10.1177/0271678X19839501.

18. Yang Z, Zhong L, Xian R, Yuan B. MicroRNA-223 regulates inflammation and brain injury via feedback to NLRP3 inflammasome after intracerebral hemorrhage. Mol Immunol. 2015;65(2):267-76. doi:10.1016/j.molimm.2014.12.018.
19. Martinez B, Peplow PV. Blood microRNAs as potential diagnostic markers for hemorrhagic stroke. Neural Regen Res. 2017;12(1):13-8. doi:10.4103/1673-5374.198965.
20. Gareev I, Yang G, Sun J, Beylerli O, Chen X, Zhang D et al. Circulating MicroRNAs as Potential Noninvasive Biomarkers of Spontaneous Intracerebral Hemorrhage. World Neurosurg. 2020;133:e369-e75. doi:10.1016/j.wneu.2019.09.016.
21. Wang Z, Fang L, Shi H, Yang Z. miR-181b regulates ER stress induced neuron death through targeting Heat Shock Protein A5 following intracerebral haemorrhage. Immunol Lett. 2019;206:1-10. doi:10.1016/j.imlet.2018.11.014.
22. Lattanzi S, Brigo F, Trinka E, Cagnetti C, Di Napoli M, Silvestrini M. Neutrophil-to-Lymphocyte Ratio in Acute Cerebral Hemorrhage: a System Review. Transl Stroke Res. 2019;10(2):137-45. doi:10.1007/s12975-018-0649-4.
23. Qi H, Wang D, Deng X, Pang X. Lymphocyte-to-Monocyte Ratio Is an Independent Predictor for Neurological Deterioration and 90-Day Mortality in Spontaneous Intracerebral Hemorrhage. Med Sci Monit. 2018;24:9282-91. doi:10.12659/MSM.911645.
