# Supplementary material for: Molecular Correlates of Hemorrhage and Edema Volumes Following Human Intracerebral Hemorrhage Implicate Inflammation, Autophagy, mRNA Splicing, and T Cell Receptor Signaling
Source: Transl Stroke Res. 2020 Nov 18;12(5):754–77. doi: 10.1007/s12975-020-00869-y (PMC8421315; doi:10.1007/s12975-020-00869-y)

Supplemental Figures

for

Molecular Correlates of Hemorrhage and Edema Volumes following Human Intracerebral Hemorrhage Implicate Inflammation, Autophagy, mRNA Splicing, and T Cell Receptor Signaling

Translational Stroke Research

Marc Durocher[1]*, Bodie Knepp[1]*, Alan Yee[1], Glen Jickling[1,2], Fernando Rodriguez[1], Kwan Ng[1], Xinhua Zhan[1], Farah Hamade[1], Eva Ferino[1], Hajar Amini[1], Paulina Carmona-Mora[1], Heather Hull[1], Bradley P. Ander[1], Frank R. Sharp[1] and Boryana Stamova[1]
*co-first authors

[1]Department of Neurology, School of Medicine, University of California at Davis, Sacramento, CA, USA.
[2]Department of Medicine, University of Alberta, Edmonton, Canada.

Corresponding Author: Boryana Stamova (bsstamova@ucdavis.edu)

Fig. S1 NLRP3 VisANT Subnetwork

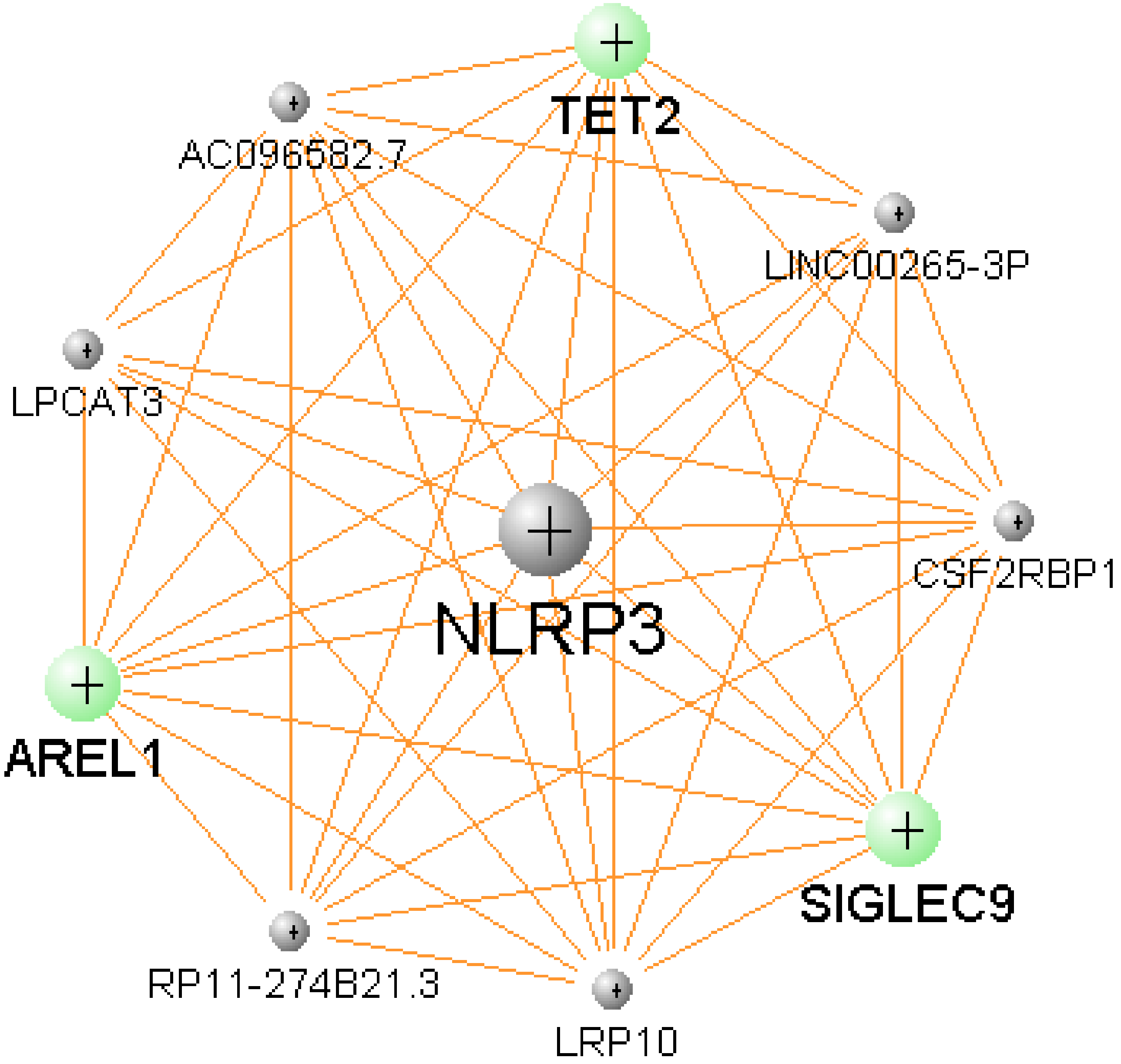

Fig. S2 NLRC4 VisANT Subnetwork

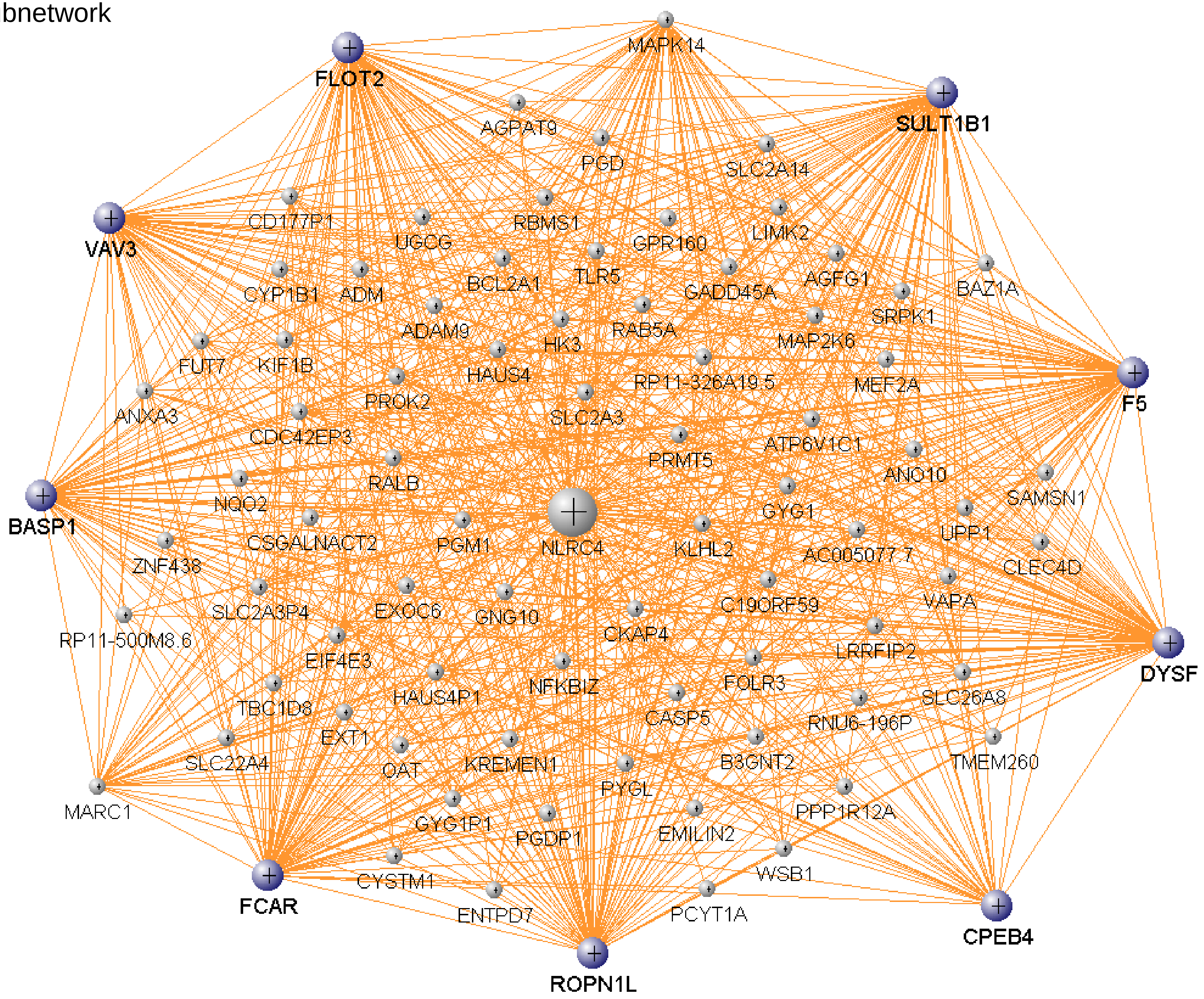

Fig. S3 NCF2 VisANT Subnetwork

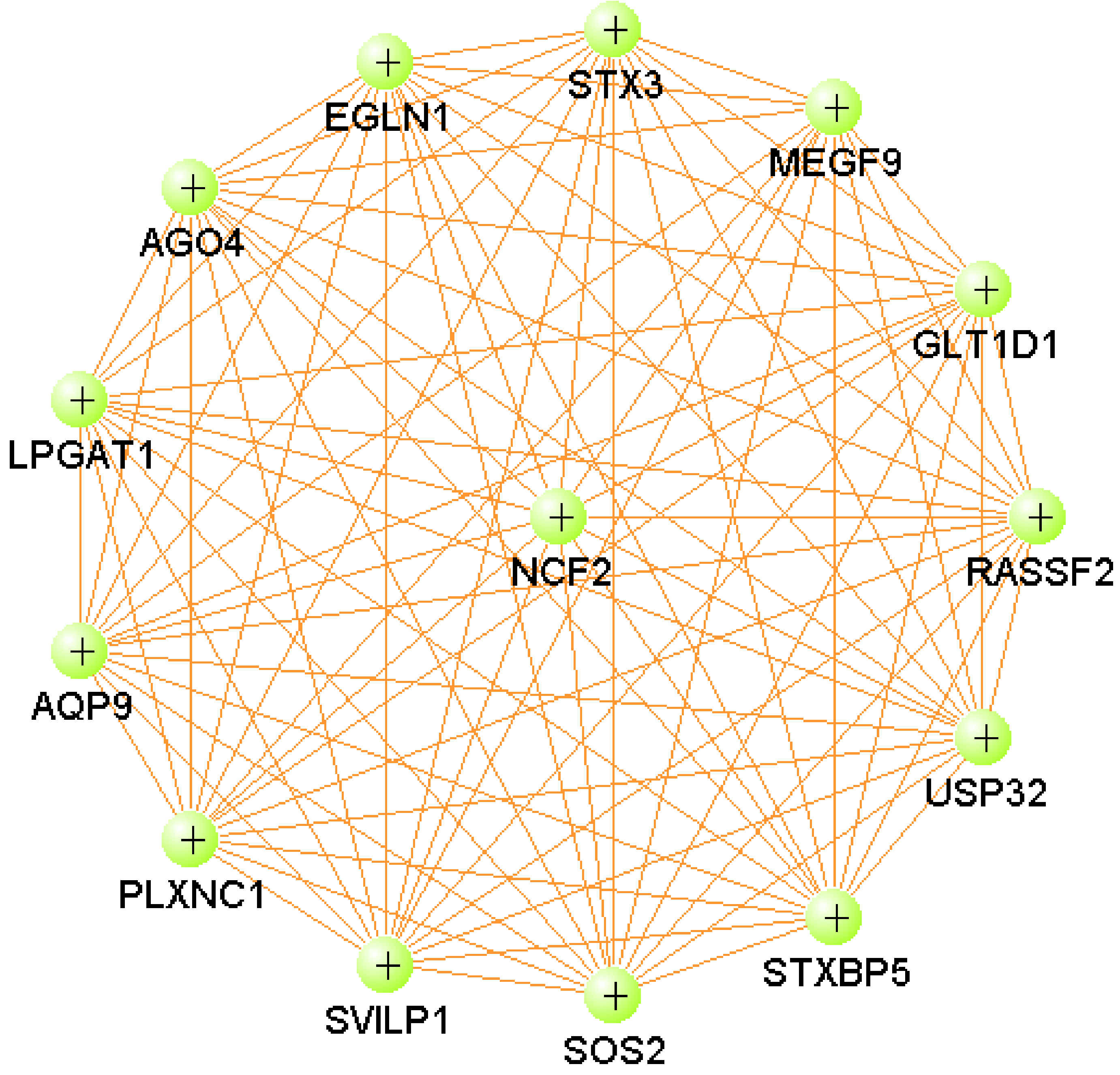

Fig. S4 CSF3R VisANT Subnetwork

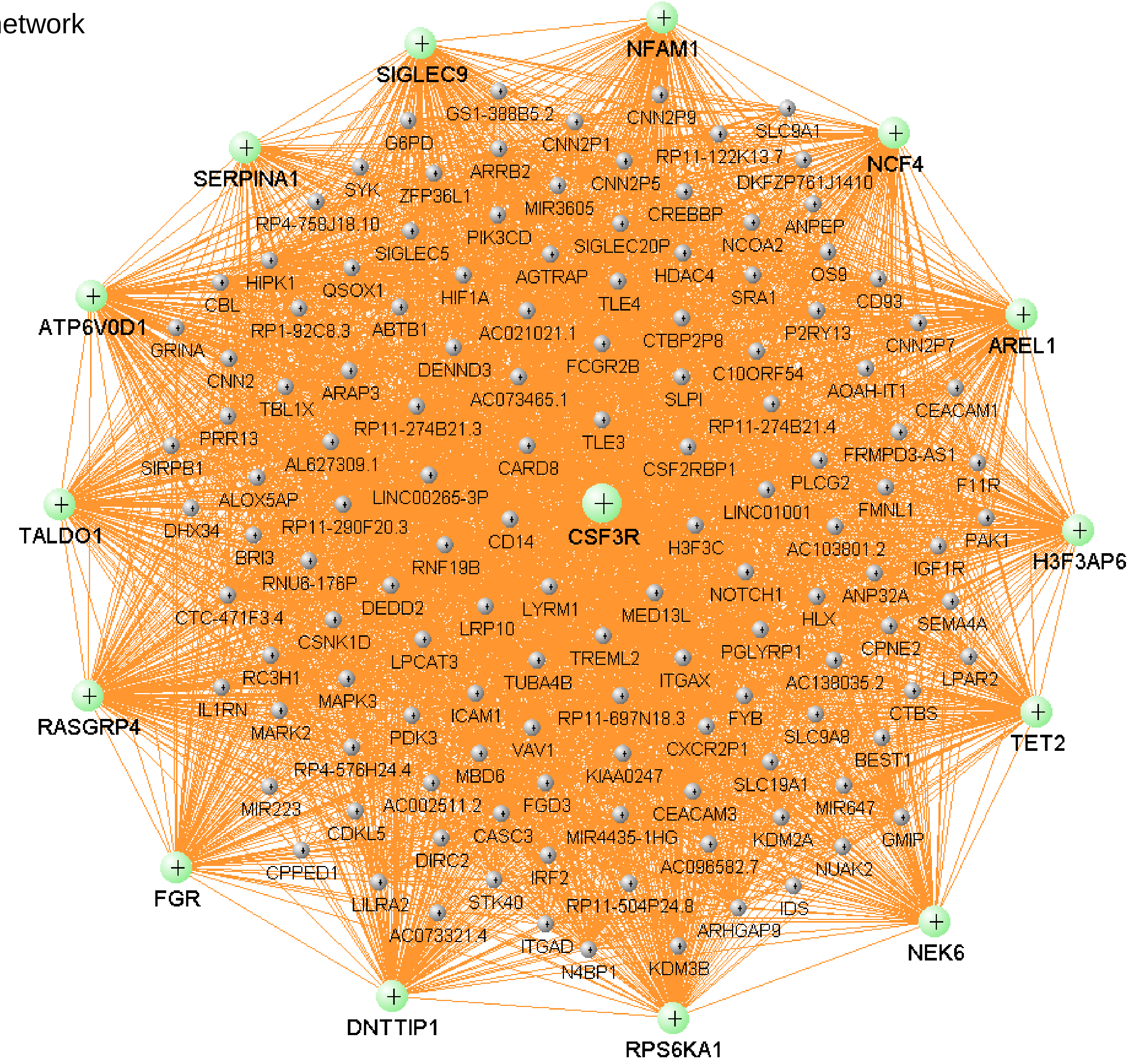

Fig. S5 Magenta T Cell Signaling Pathway using ICH correlations

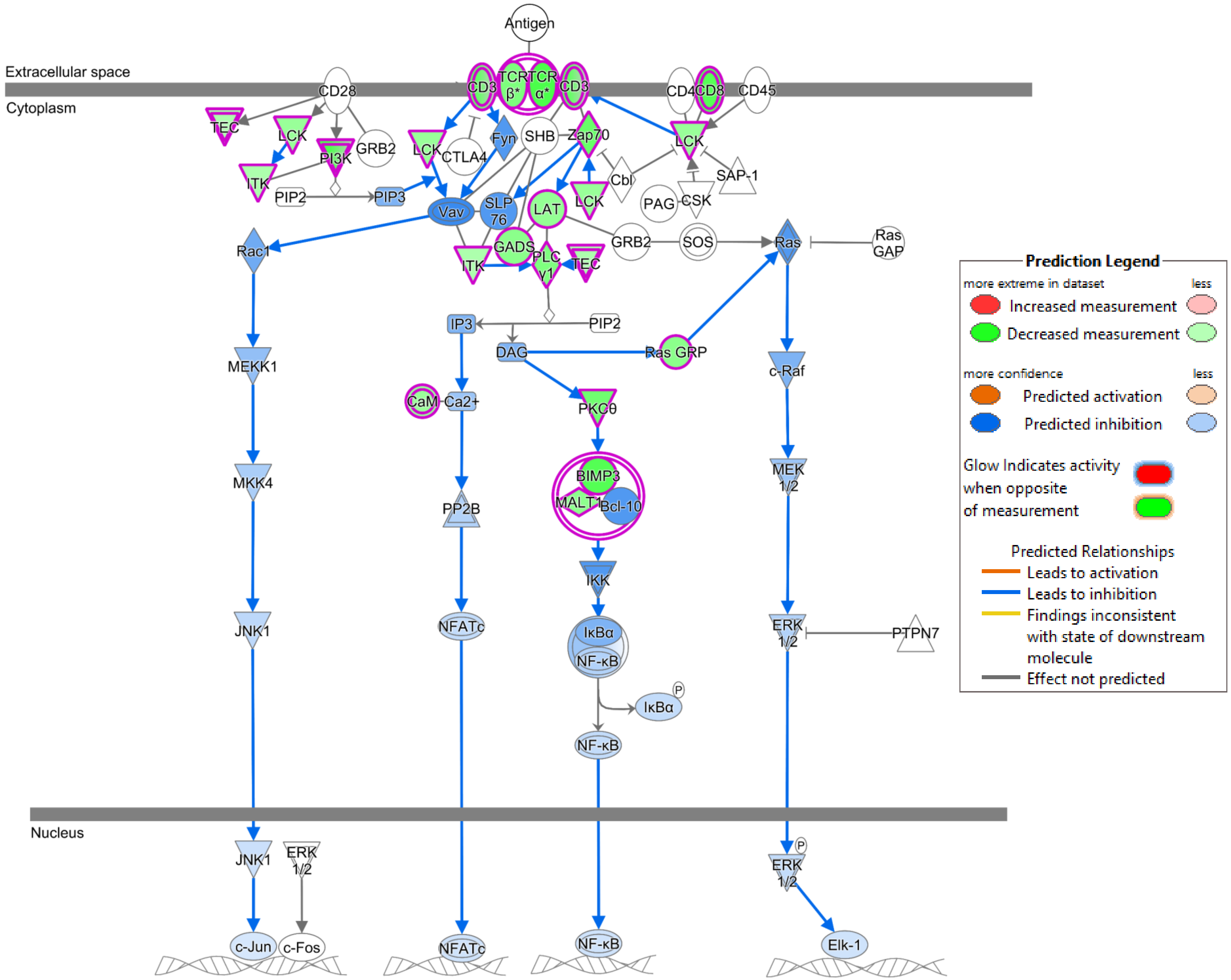

Fig. S6 TRAJ36 VisANT Subnetwork

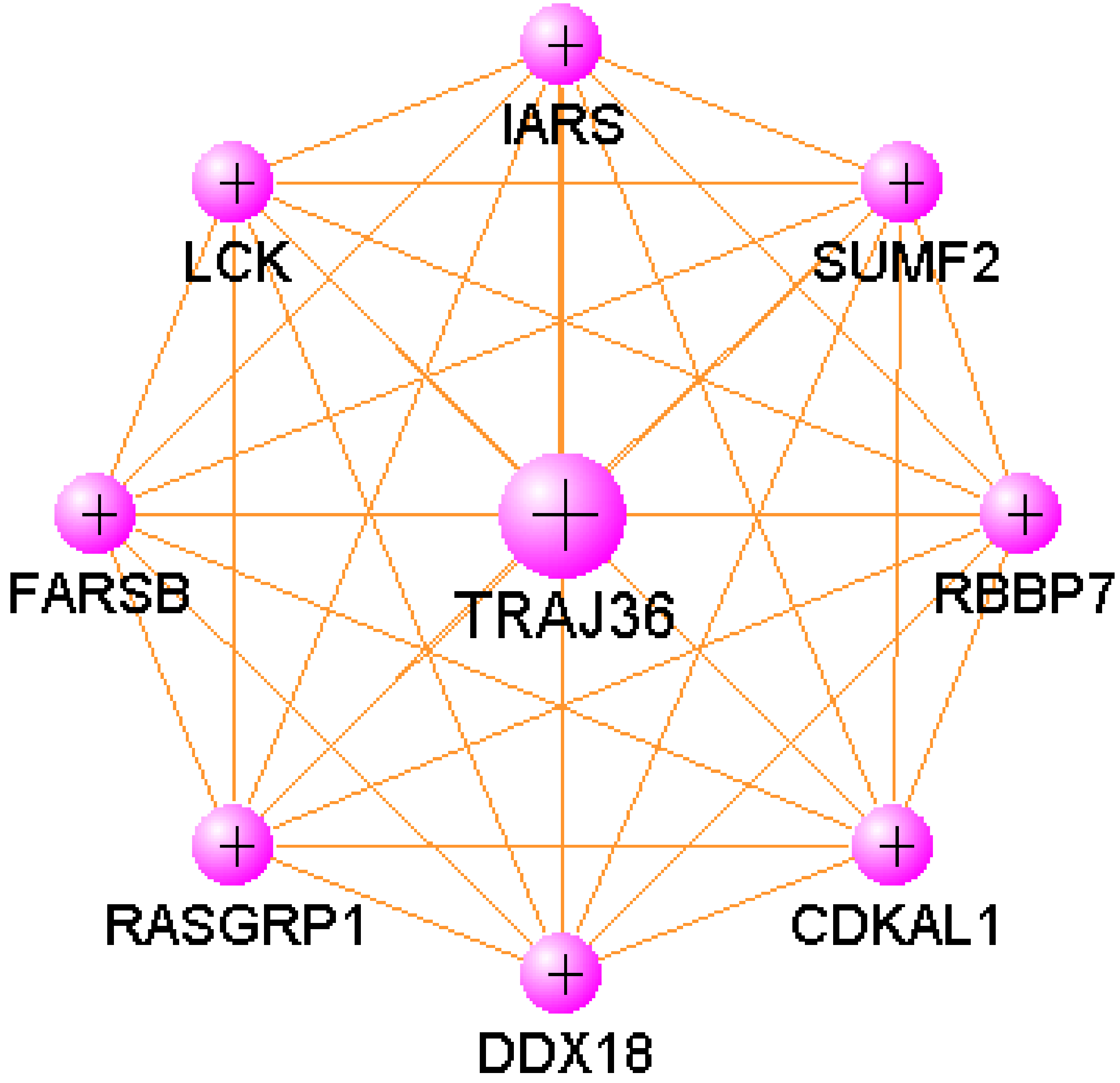

Fig. S7 ITK VisANT Subnetwork

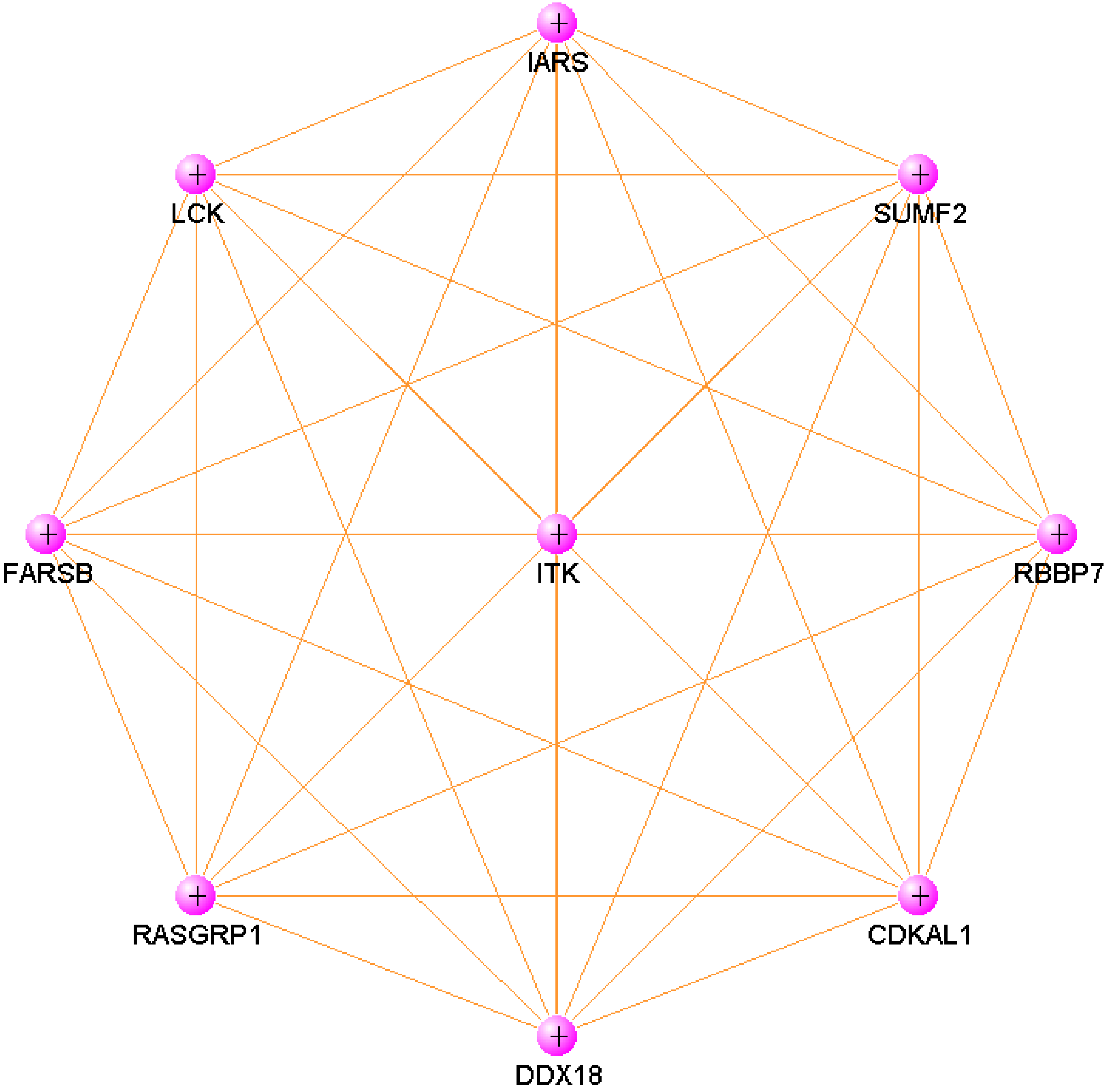

Fig. S8 LCK VisANT Subnetwork

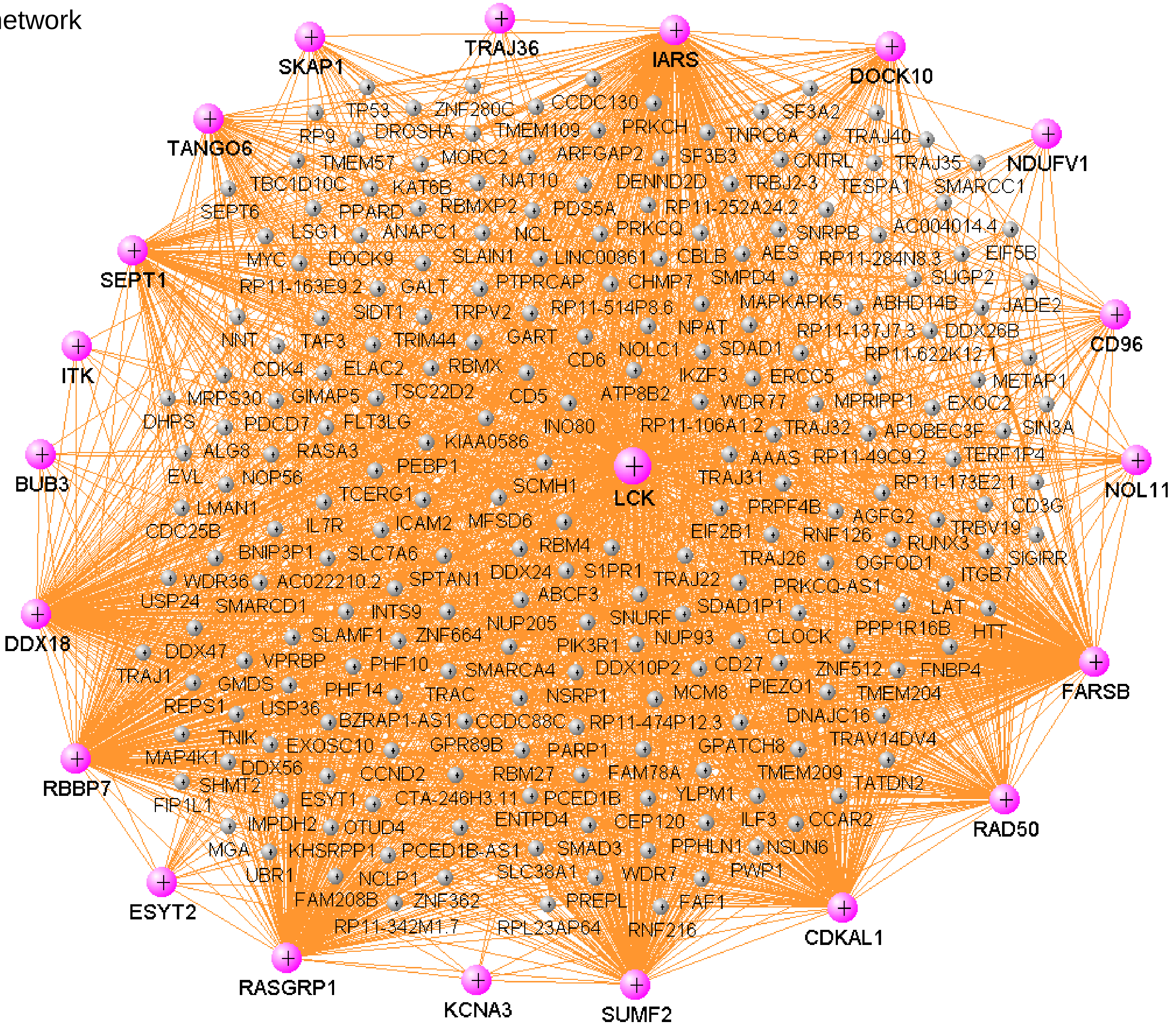

Fig. S9 S1PR1 VisANT Subnetwork

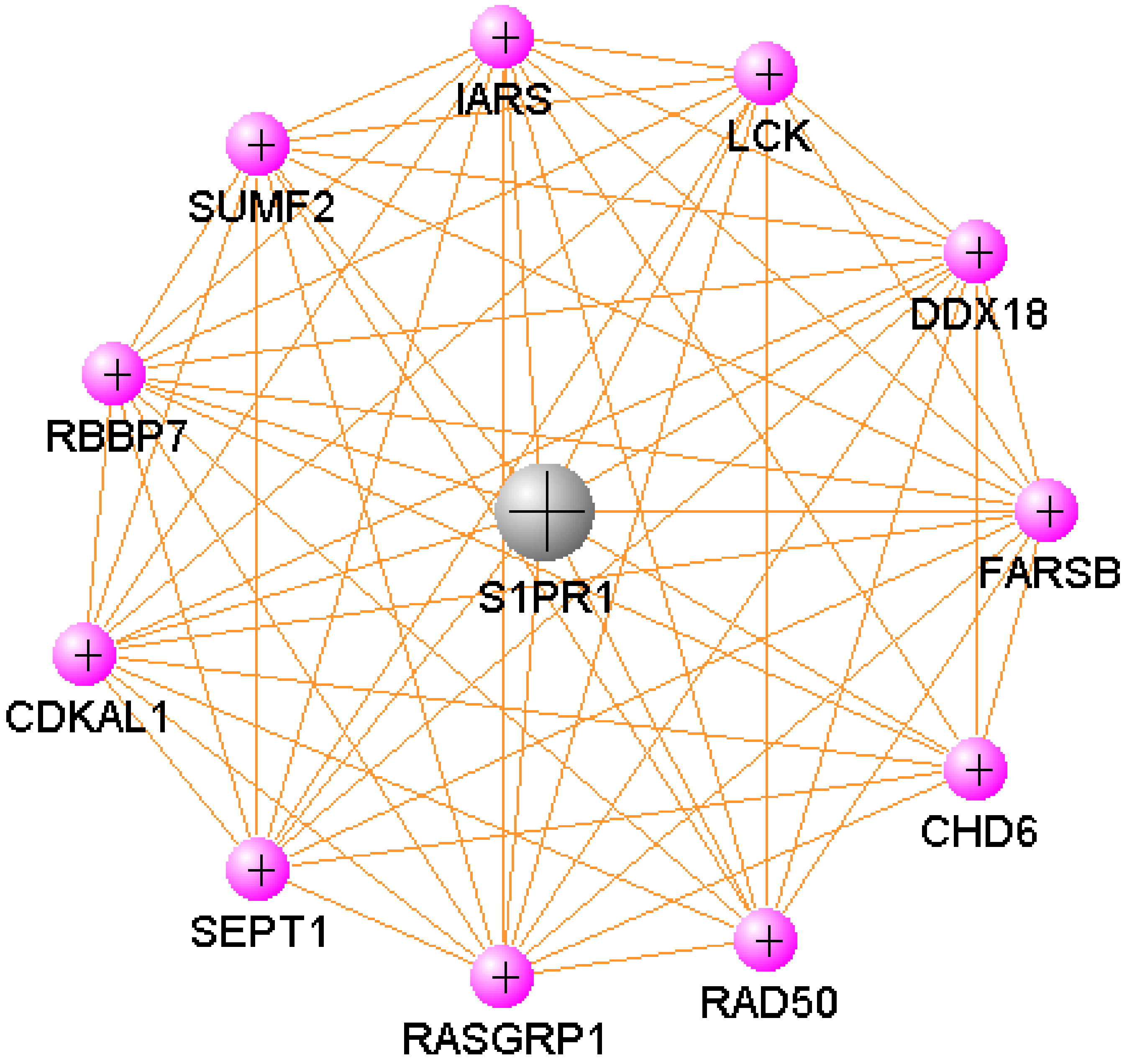

Fig. S10 SKAP1 VisANT Subnetwork

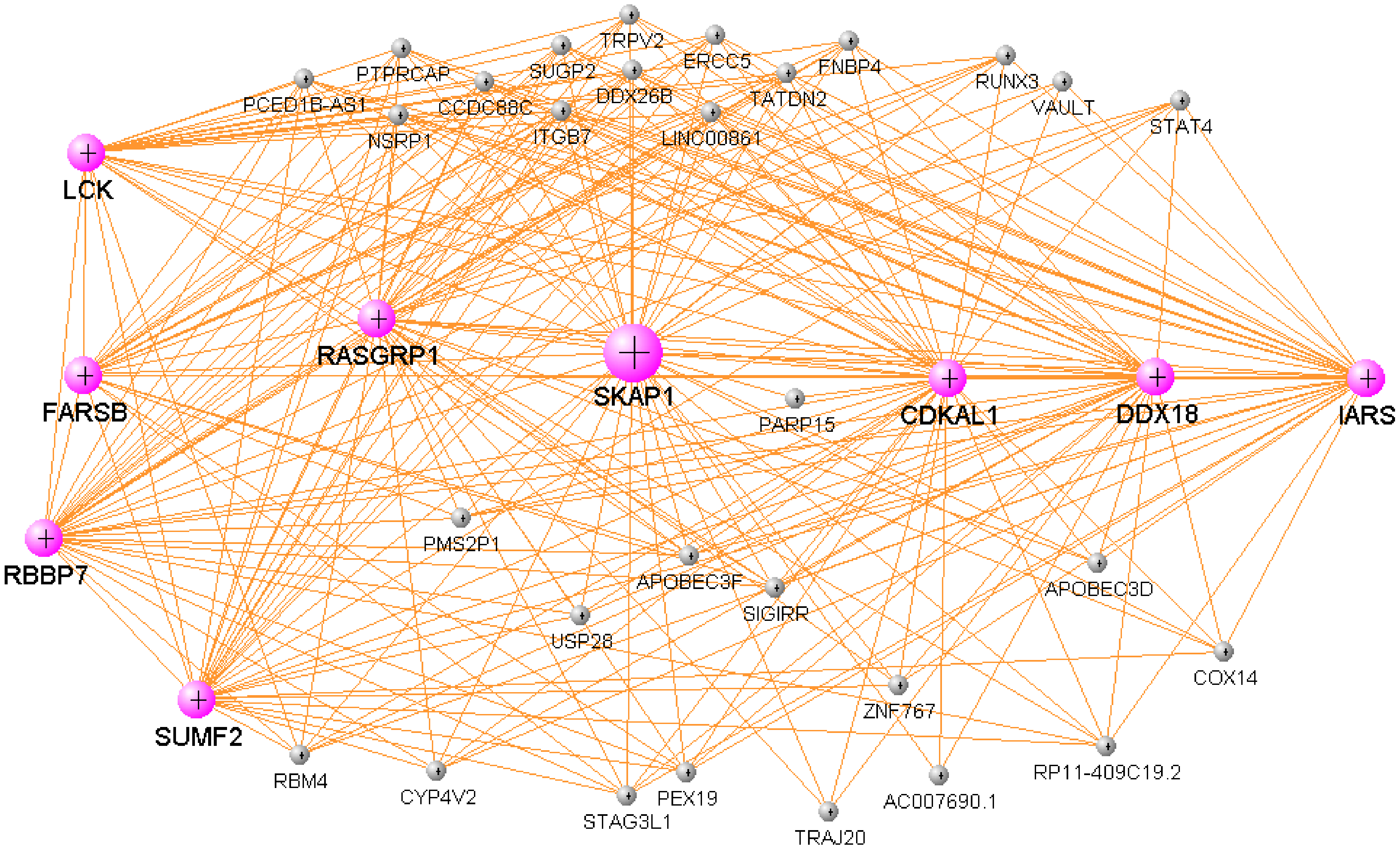

Fig. S11 ICH-aPHE Plot and Correlation

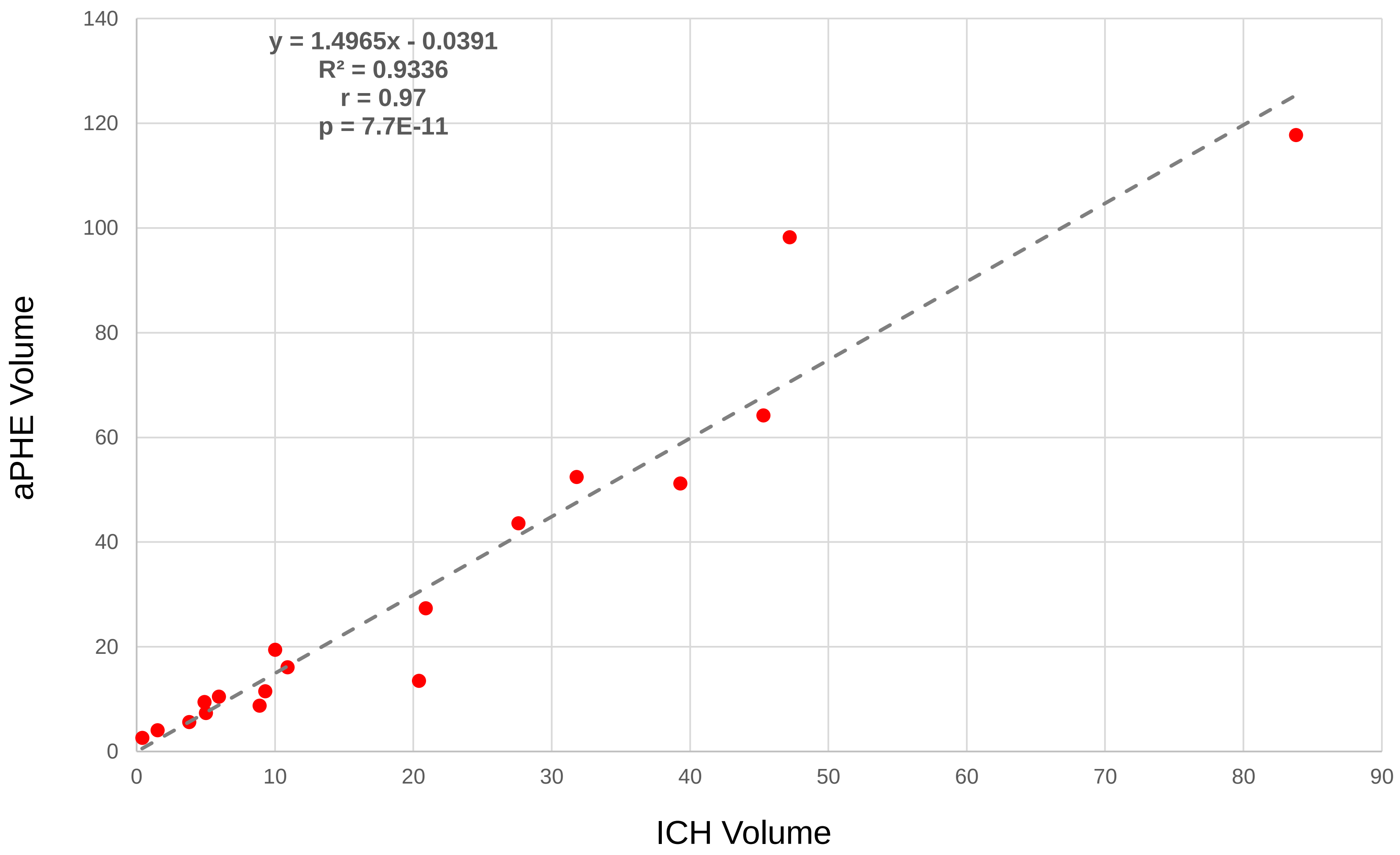

Fig. S12A ICH-rPHE Plot and Correlation

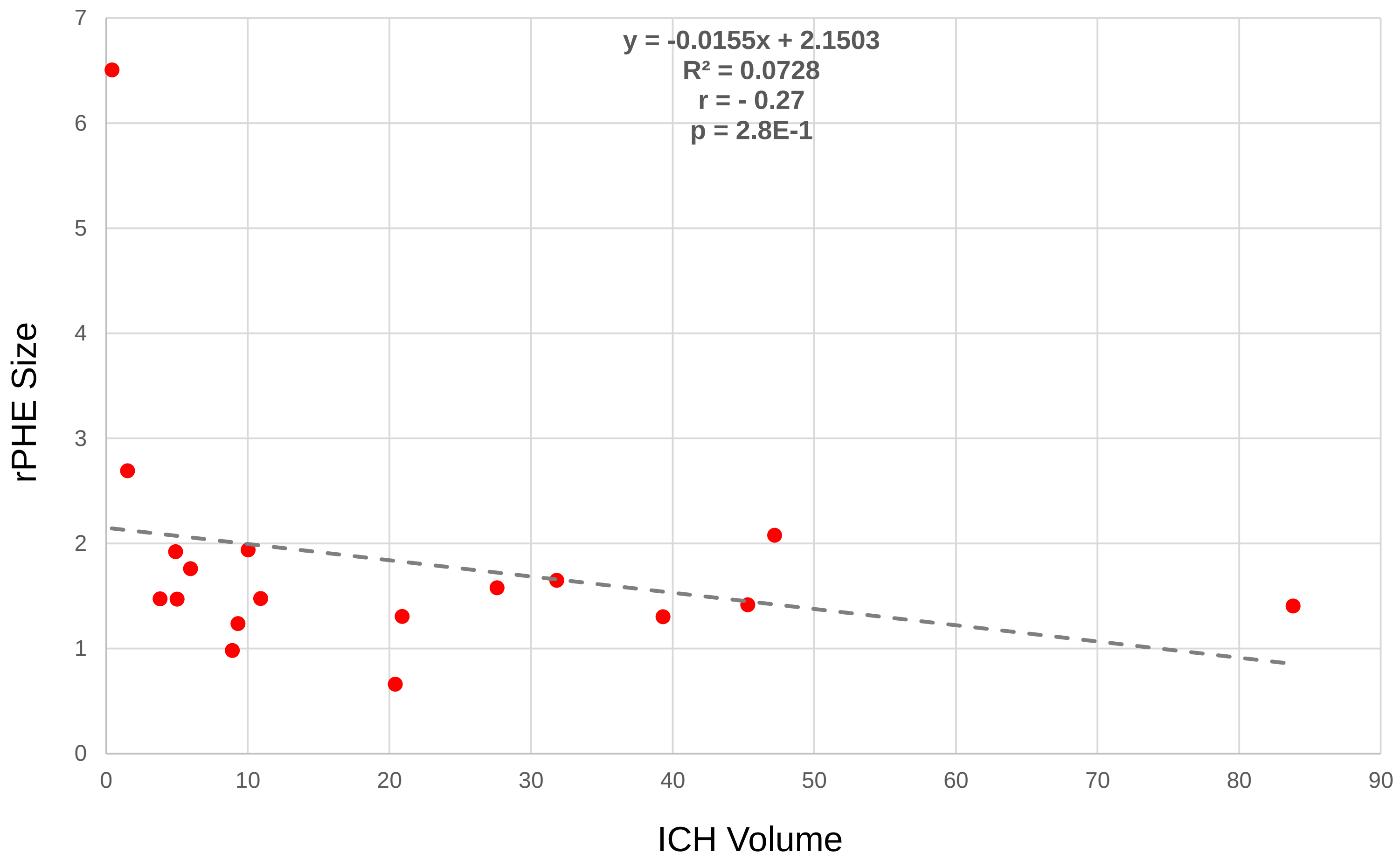

Fig. S12B aPHE-rPHE Plot and Correlation

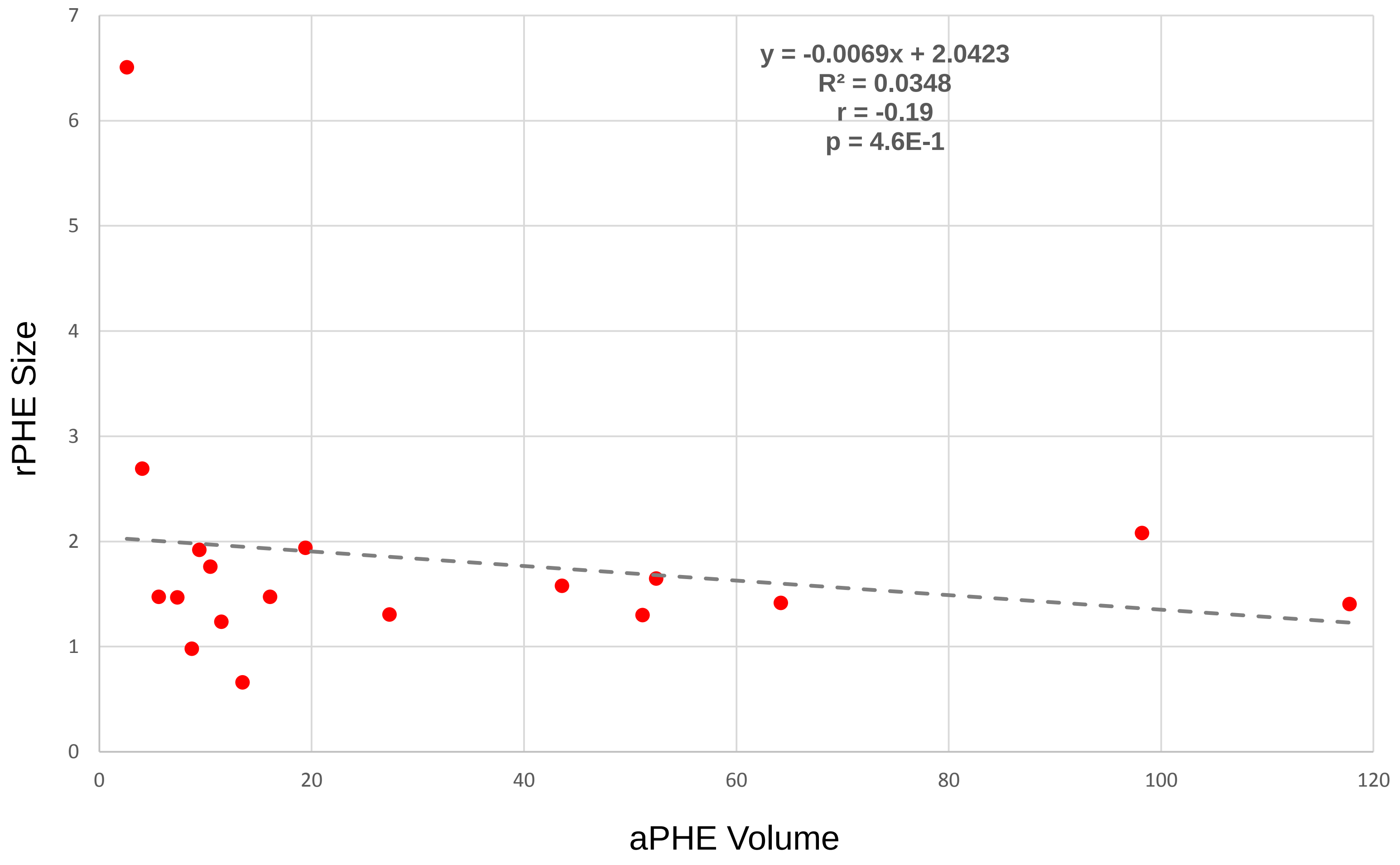

Fig. S13 AQP9 VisANT Subnetwork

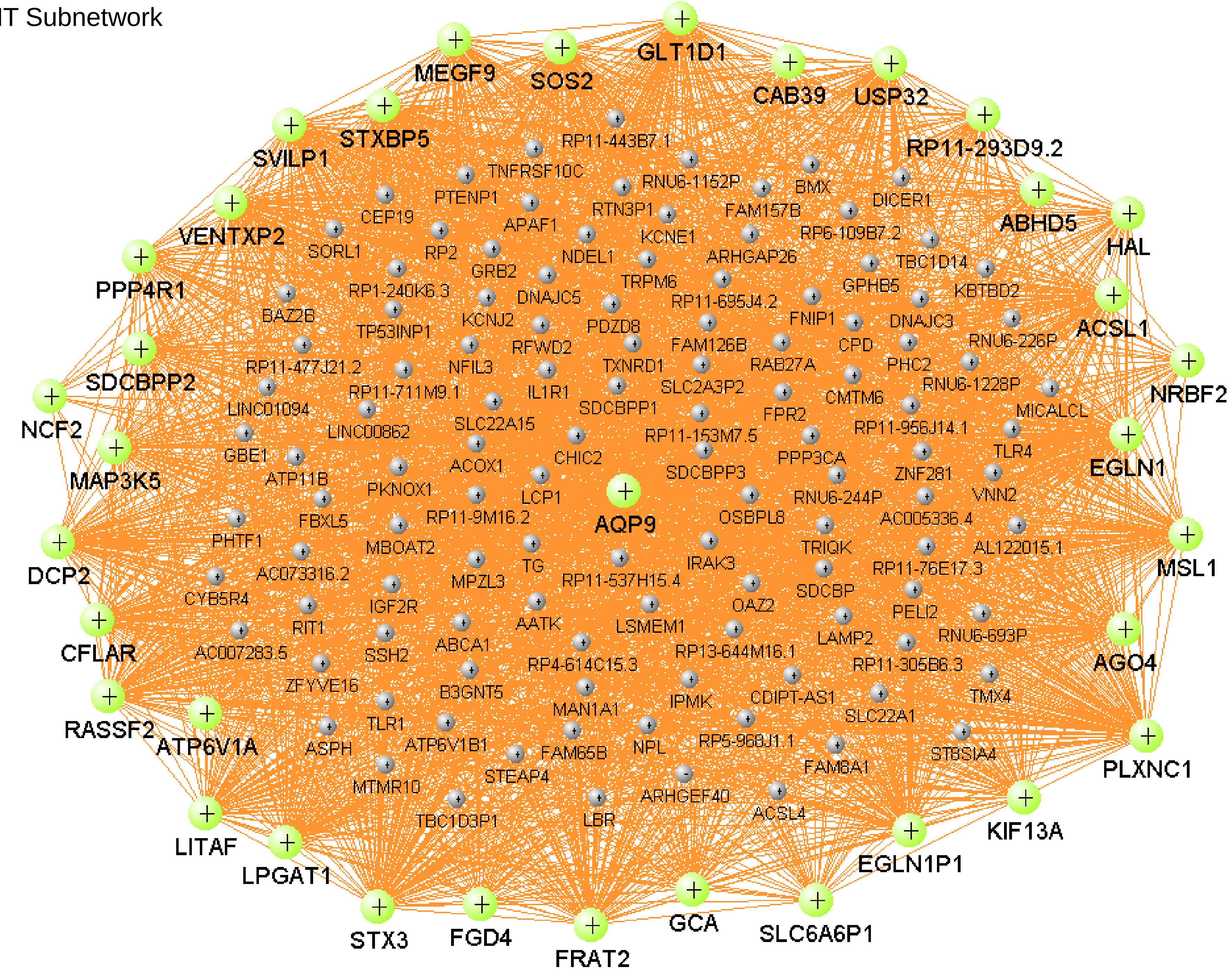

Fig. S14 ICH Hub Enrichment in Immune and Leukocytic Processes

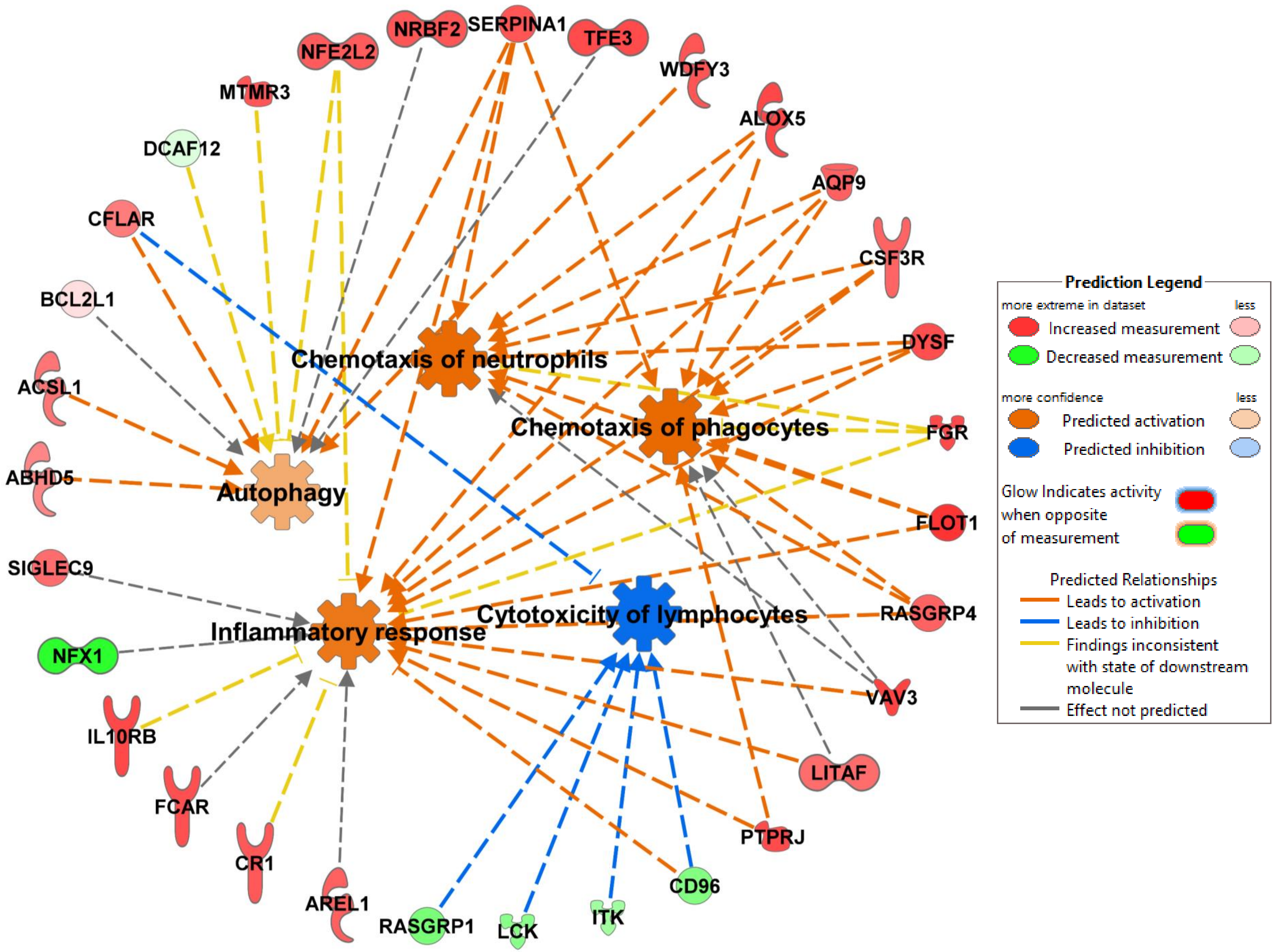

Fig. S15 NF-kB Signaling pathway in ICH per-gene analysis

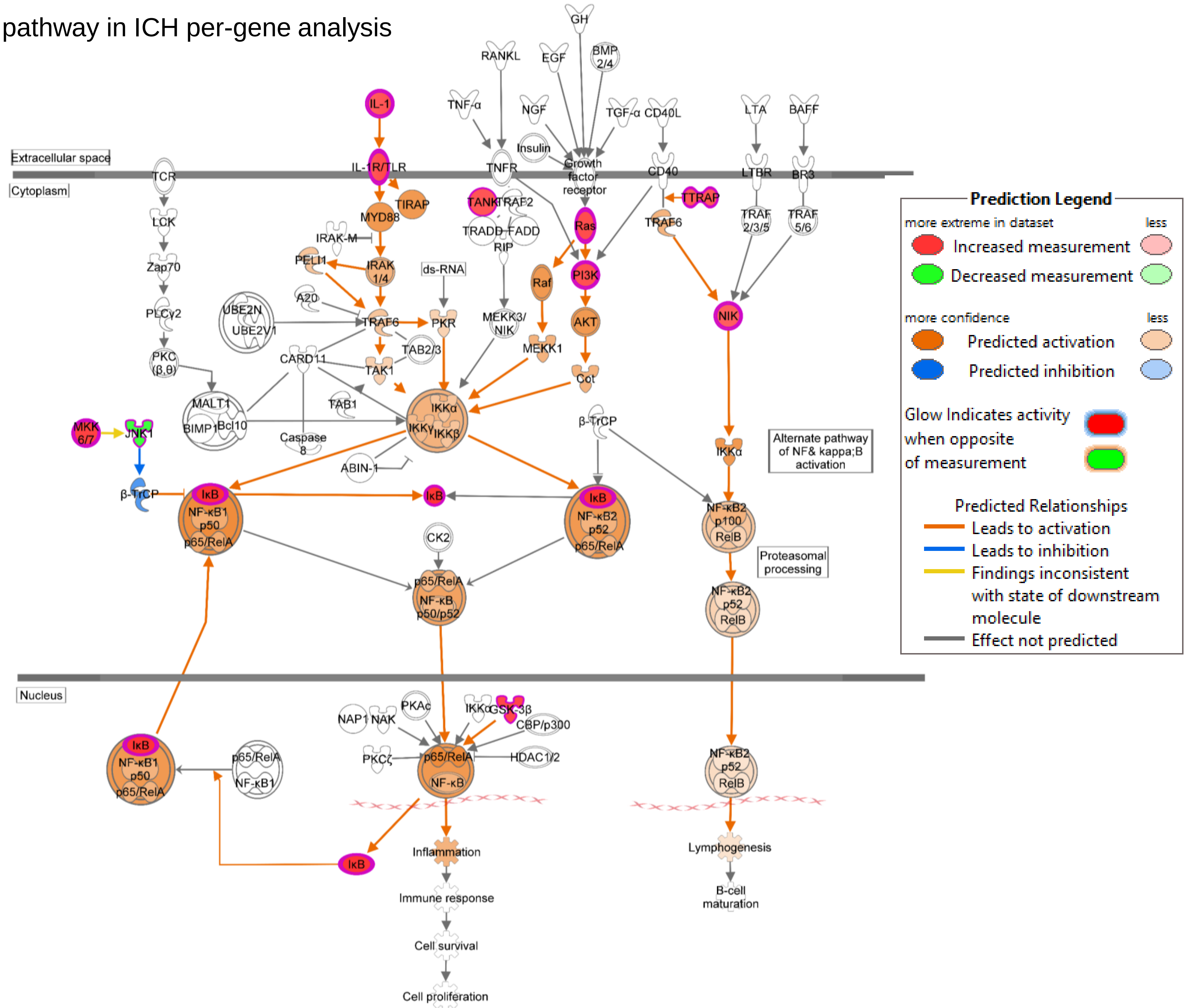

Fig. S16 VCAN VisANT Subnetwork

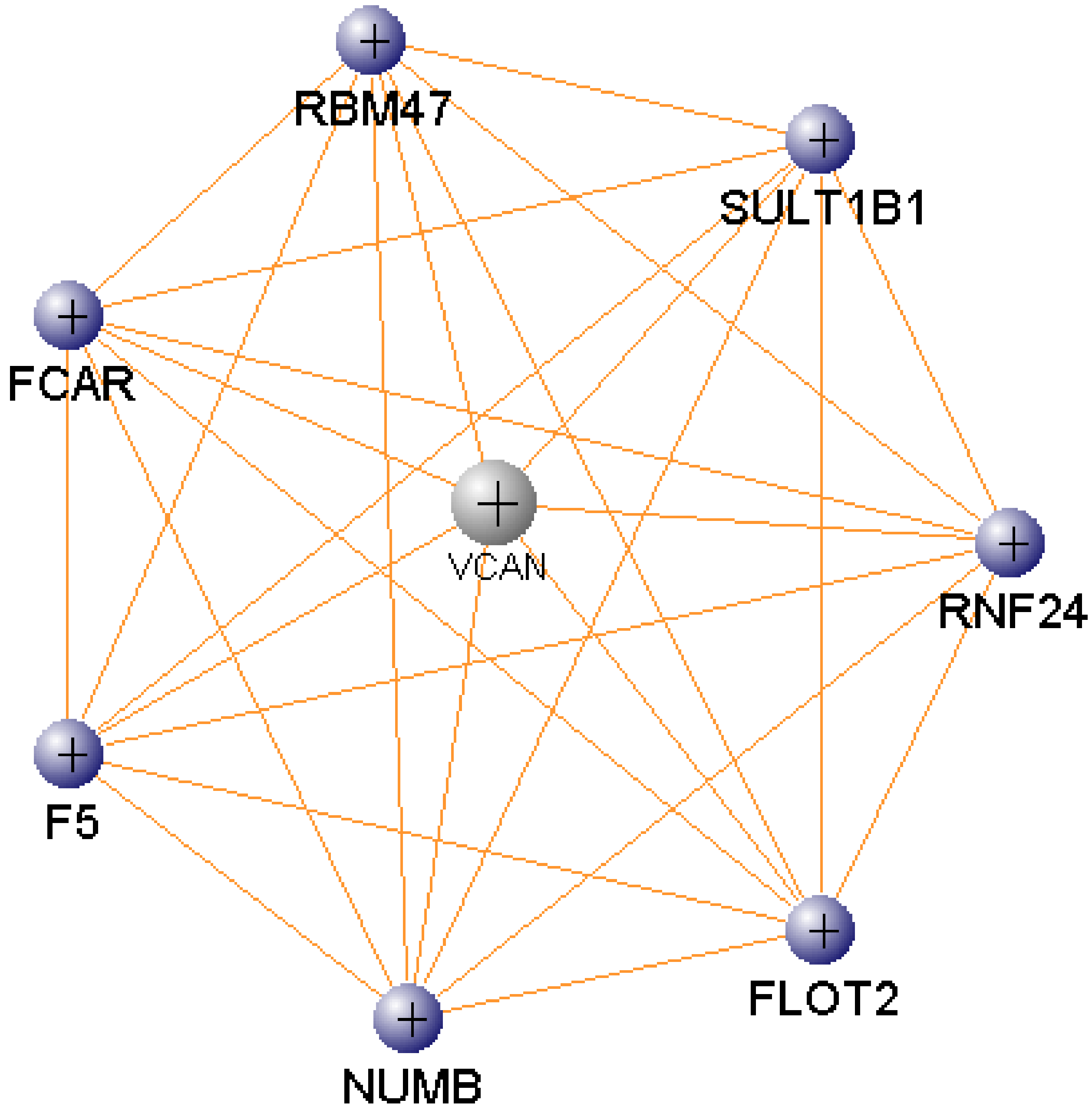

Fig. S17 HMOX1 VisANT Subnetwork

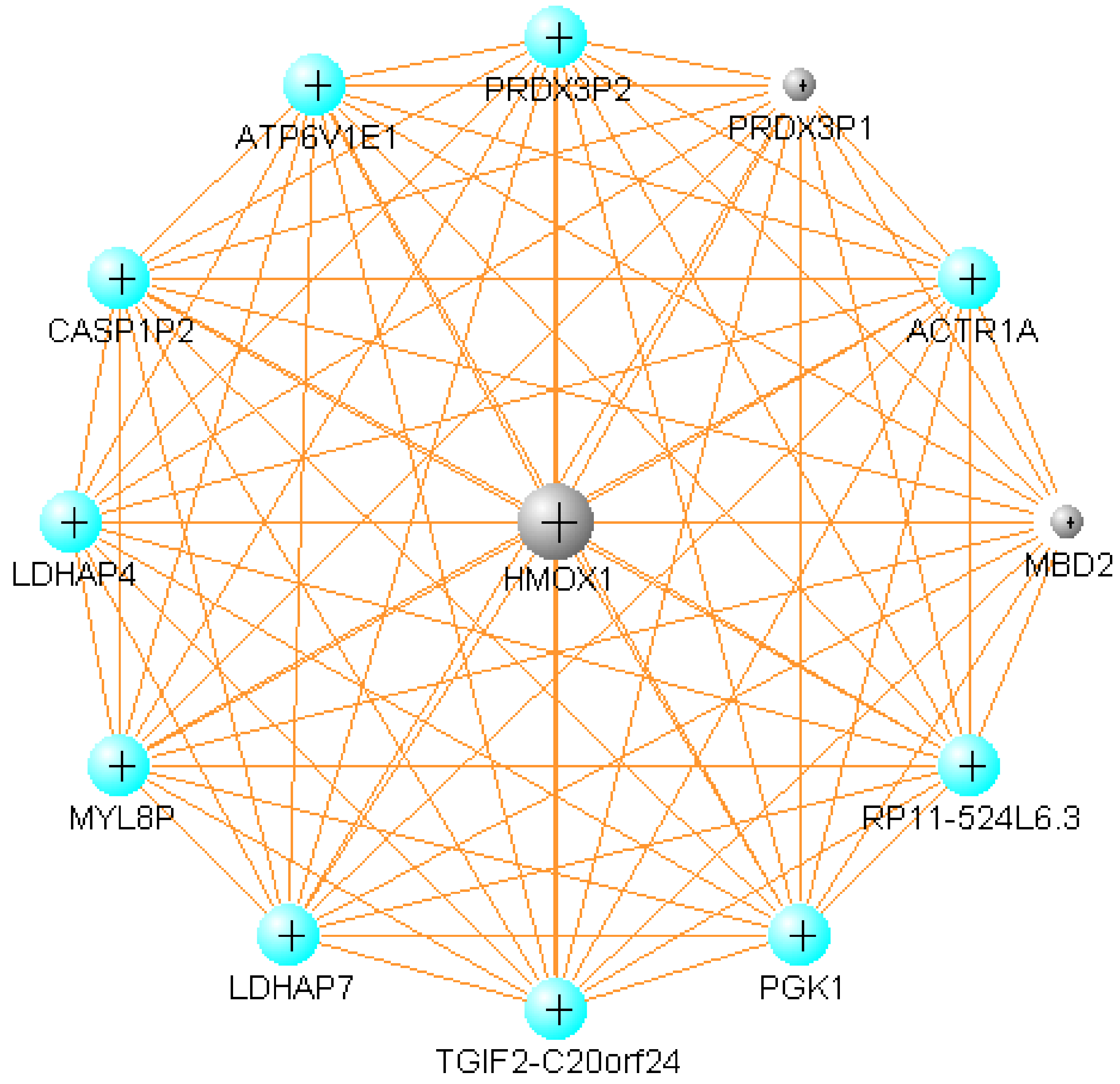

Fig. S18 RASGRP1 VisANT Subnetwork

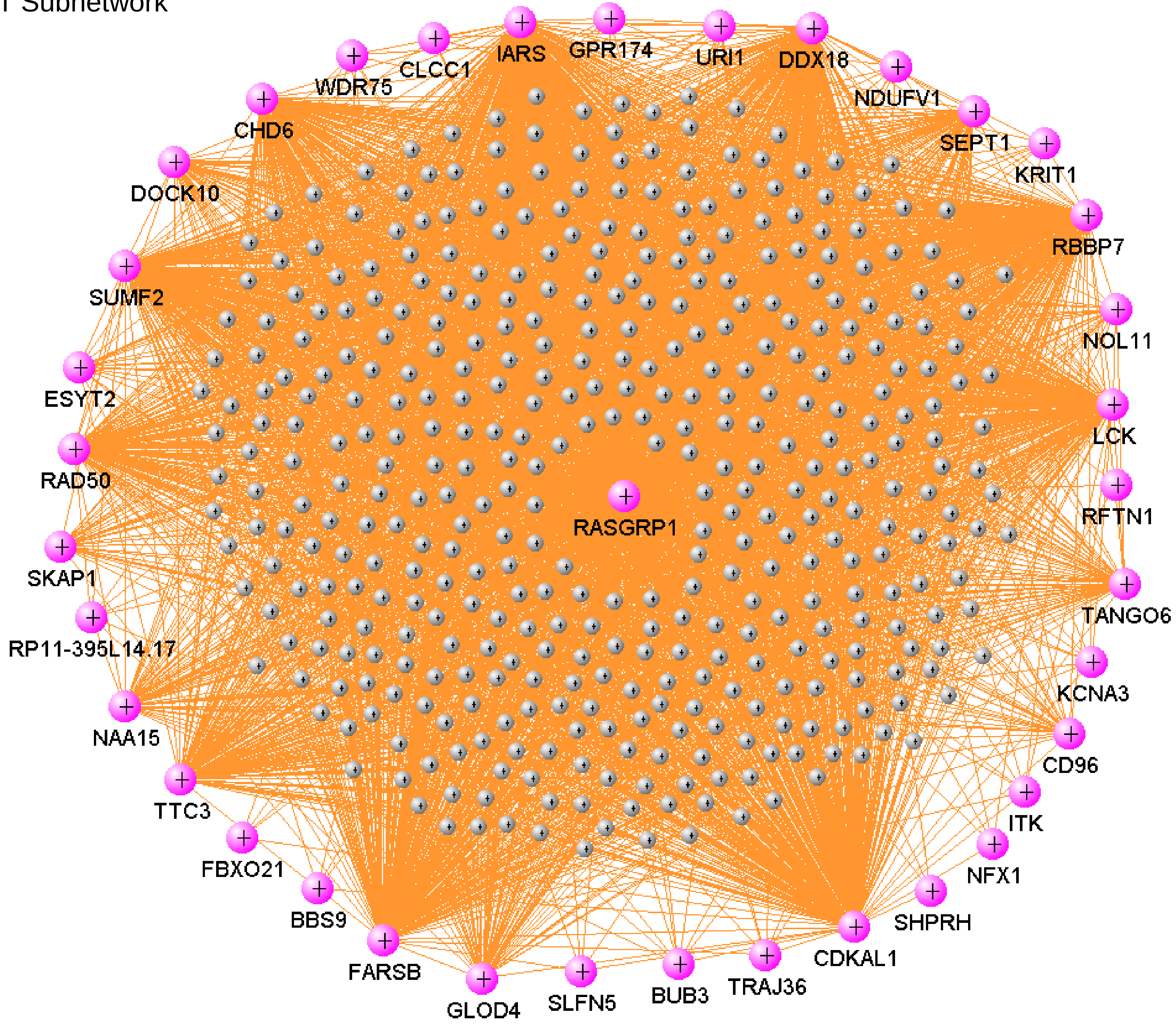

Fig. S19 RAD50 VisANT Subnetwork

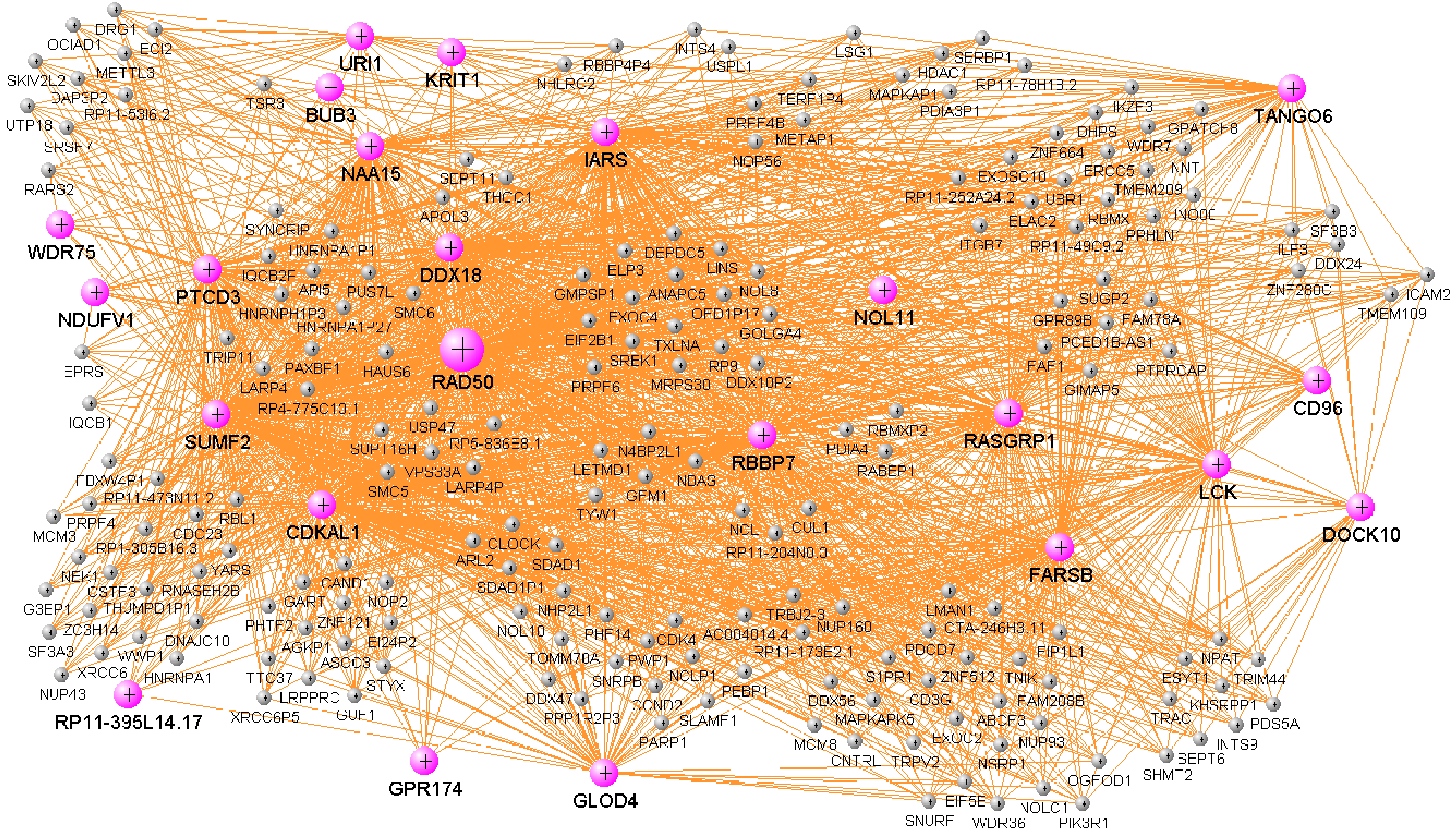

Fig. S20 KCNA3 VisANT Subnetwork

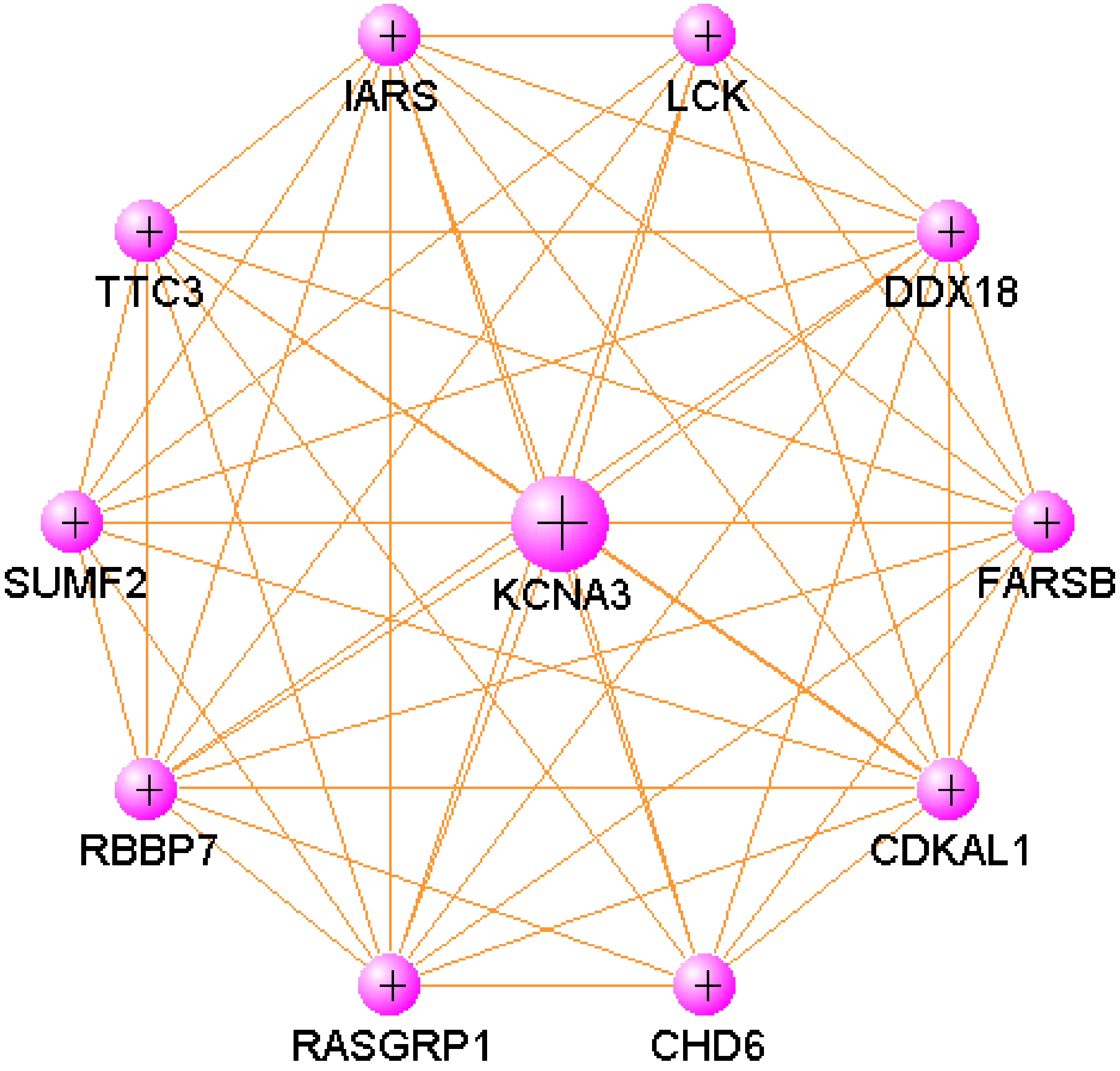

Fig. S21 FBXO21 VisANT Subnetwork

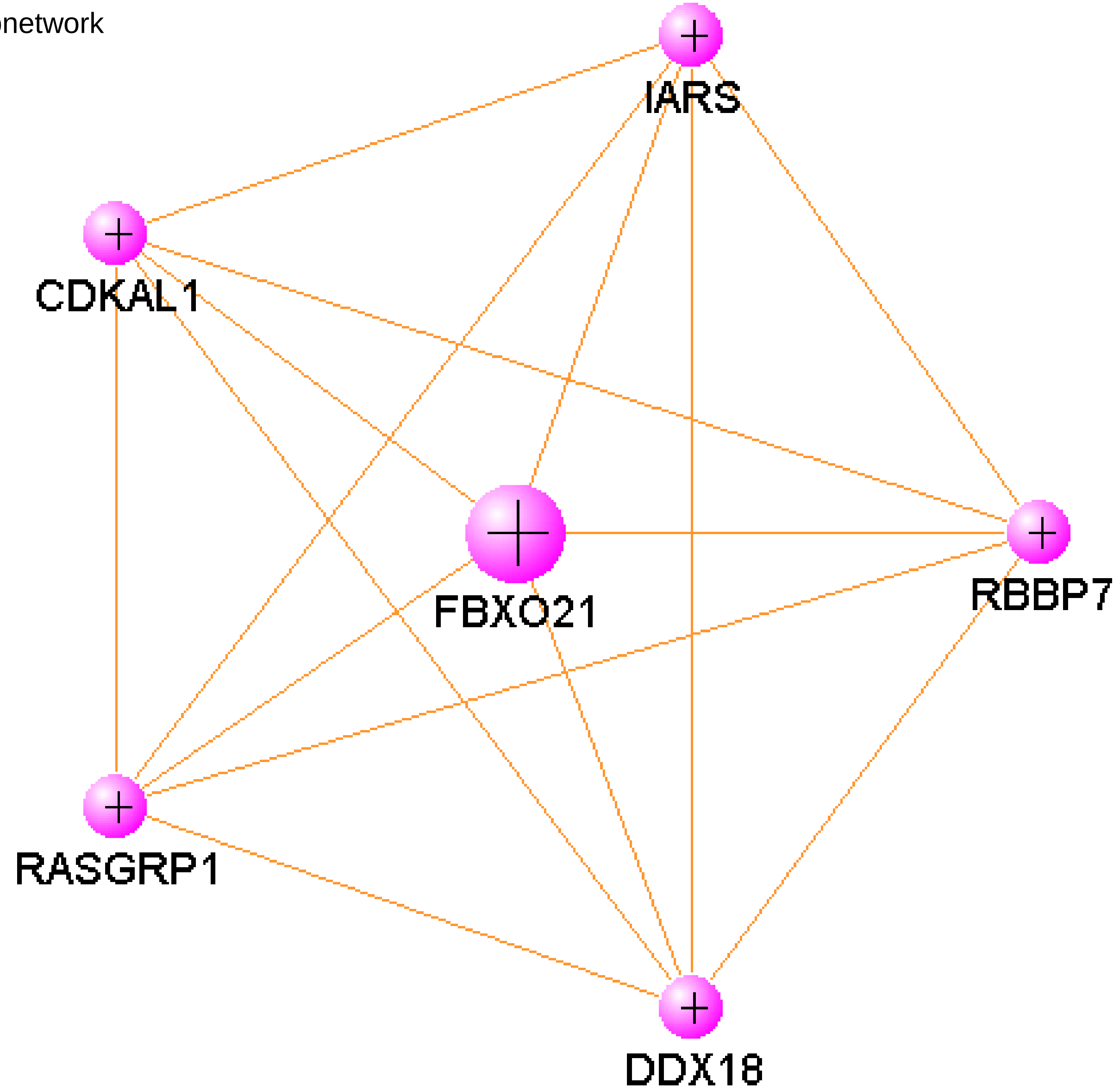

Supplement: Supplementary file 2 — Supplemental Figures (PDF 2218 kb) [file 12975_2020_869_MOESM2_ESM.pdf]
