# Supplementary material for: Molecular Correlates of Hemorrhage and Edema Volumes Following Human Intracerebral Hemorrhage Implicate Inflammation, Autophagy, mRNA Splicing, and T Cell Receptor Signaling
Source: Transl Stroke Res. 2020 Nov 18;12(5):754–77. doi: 10.1007/s12975-020-00869-y (PMC8421315; doi:10.1007/s12975-020-00869-y)
Supplement: Supplementary file 4 — Captions for Supplemental Material (PDF 82.4 kb) [file 12975_2020_869_MOESM4_ESM.pdf]

ESM 1 Supplemental Methods and Discussion

Detailed materials and methods as well as additional discussion of the findings of this study.

ESM 2 Supplemental Figures

Fig. S1 NLRP3 network in the LightGreen module. Genes colored in light green are hub genes

Fig. S2 NLRC4 network in the MidnightBlue module. Genes colored in midnight blue are hub genes

Fig. S3 NCF2 network in the GreenYellow module. Genes colored in green yellow are hub genes

Fig. S4 CSF3R network in the LightGreen module. Genes colored in light green are hub genes

Fig. S5 T Cell Signaling Pathway enrichment in Magenta module genes shown with predicted activation (orange) and suppression (blue) of associated molecules and relationships. Genes and complexes outlined in purple were present in the Magenta module, and their green shading corresponds to the strength of their negative correlation with ICH volume

Fig. S6 TRAJ32 network in the Magenta module. Genes colored in magenta are hub genes

Fig. S7 ITK network in the Magenta module. Genes colored in magenta are hub genes

Fig. S8 LCK network in the Magenta module. Genes colored in magenta are hub genes

Fig. S9 S1PR1 network in the Magenta module. Genes colored in magenta are hub genes

Fig. S10 SKAP1 network in the Magenta module. Genes colored in magenta are hub genes

Fig. S11 Correlation of patients' aPHE and ICH volumes

Fig. S12 Correlation of patients' rPHE size and ICH volume (A). Correlation of patients' rPHE size and aPHE volume (B)

Fig. S13 AQP9 network in the GreenYellow module. Genes colored in green yellow are hub genes

Fig. S14 Correlations of hub genes in 5 modules significant to ICH predicted the activation of the inflammatory response, autophagy, and the chemotaxis of neutrophils and phagocytes. The cytotoxicity of lymphocytes was predicted to be suppressed

Fig. S15 Per Gene ICH enrichment in NF-κB Signaling Pathway with predicted activation (orange) and suppression (blue) of associated molecules and relationships. Genes and complexes outlined in purple were present in the Magenta module. Green shading represents the strength of the negative correlation to ICH volume, while red shading represents the strength of the positive correlation to ICH volume

Fig. S16 VCAN network in the MidnightBlue module. Genes colored in midnight blue are hub genes

Fig. S17 HMOX1 network in the Cyan module. Genes colored in cyan are hub genes

Fig. S18 RASGRP1 network in the Magenta module. Genes colored in magenta are hub genes

Fig. S19 RAD50 network in the Magenta module. Genes colored in magenta are hub genes

Fig. S20 KCNA3 network in the Magenta module. Genes colored in magenta are hub genes

Fig. S21 FBXO21 network in the Magenta module. Genes colored in magenta are hub genes

ESM 3 Supplemental Tables

Table S1 Genes significantly correlated with the volumetric measures (p<0.005, |r|>0.6) in the per gene analyses

Table S2 IPA Canonical Pathway Enrichment (BH p<0.05) for per gene, significant modules, and hub gene lists

Table S3 DAVID Gene Ontology (FDR p<0.05) for per gene, significant modules, and hub gene lists

Table S4 Hypergeometric Probability Testing for per gene, significant modules, and hub gene lists

Table S5 Gene Assignments for 7 significant modules

Table S6 Module Significance with respect to volumetric measures, vascular risk factors, and other clinical and demographic characteristics

Table S7 IPA Disease and Function Enrichment (p<0.05) for per gene, significant modules, and hub gene lists
